# "What else are you worried about?" – Integrating textual responses into quantitative social science research

**DOI:** 10.1371/journal.pone.0182156

**Published:** 2017-07-31

**Authors:** Julia M. Rohrer, Martin Brümmer, Stefan C. Schmukle, Jan Goebel, Gert G. Wagner

**Affiliations:** 1 Department of Psychology, University of Leipzig, Leipzig, Germany; 2 German Institute for Economic Research, Berlin, Germany; 3 Max Planck Institute for Human Development, Berlin, Germany; 4 AKSW, Institute for Applied Informatics, University of Leipzig, Leipzig, Germany; 5 Berlin University of Technology, Berlin, Germany; University of Bedfordshire, UNITED KINGDOM

## Abstract

Open-ended questions have routinely been included in large-scale survey and panel studies, yet there is some perplexity about how to actually incorporate the answers to such questions into quantitative social science research. Tools developed recently in the domain of natural language processing offer a wide range of options for the automated analysis of such textual data, but their implementation has lagged behind. In this study, we demonstrate straightforward procedures that can be applied to process and analyze textual data for the purposes of quantitative social science research. Using more than 35,000 textual answers to the question “What else are you worried about?” from participants of the German Socio-economic Panel Study (SOEP), we (1) analyzed characteristics of respondents that determined whether they answered the open-ended question, (2) used the textual data to detect relevant topics that were reported by the respondents, and (3) linked the features of the respondents to the worries they reported in their textual data. The potential uses as well as the limitations of the automated analysis of textual data are discussed.

## Introduction

Open-ended questions such as “Would you like to add anything?”, “Is there anything else you would like to tell us?”, “Please tell us anything you think is important” are commonly used as complements in surveys that otherwise rely heavily on closed-ended questions [1]. However, to this day–decades after the collection of such textual answers began–routines have yet to be established for analyzing the unstandardized textual answers–so-called free texts–and for integrating them into quantitative social science research.

On the other hand, the in-depth analysis of textual data such as diaries, discourses, or transcripts of interviews is an established part of qualitative research. The so-called Qualitative Content Analysis (QCA, see [2] for a brief overview and examples of its application) offers a range of techniques to approach the content of a text on different levels, from the gist of the text to subtle references that can be understood only in the broader context of current events and public discourses. Content analysis itself covers a wide range of different strategies. For example, researchers can derive categories of interest from the data itself, from theory, or from prior research. Researchers are also able to focus on the keywords that are identified from the underlying context [3].

While these analytical strategies might aim to fulfill quality criteria that are well known to quantitative researchers, such as reliability and validity [2], their utility is limited when considering the types of answers submitted as answers to open-ended questions on surveys. Open-ended questions on surveys typically generate a large number of short texts, in contrast to the small numbers of long and comprehensive texts that are routinely analyzed in QCA. Respondents of large surveys often provide only one or a handful of words in their answers because of (1) the narrow phrasing of the questions, (2) a lack of motivation to answer exhaustively, (3) space limitations on the questionnaire, or (4) time pressure in the interview situation due to the interest of the interviewer to complete the interview quickly. This brevity might make the work that is necessary to apply a thorough QCA appear excessive: The answers are short, but the number of respondents is very high.

O’Cathain and Thomas [1] fittingly characterized the data generated by open-ended questions as neither strictly qualitative nor strictly quantitative, a status the two authors describe as “uncomfortable.” As a reaction to this intermediate state, quantitative researchers who decide to use such textual data often use a strategy of low-key “quantitizing” to solve the issue in the manner in which they are accustomed: Texts are manually replaced by numeric codes representing categories that are supposed to be relevant [4]. Quantitizing is also used in qualitative and mixed methods research and is guided by a number of assumptions and judgments that might be glossed over in the process [4] or, in the case of quantitative research, not even stated explicitly.

But even low-key quantitizing is labor intensive when the number of respondents is very high as it is when large-sample surveys are used. In this case, automated text analysis offers an effective way to tackle the data. Natural language processing (NLP) is a multidisciplinary field in which the interaction between human language and computers is explored. Tools that have been developed in this domain allow several steps of text analysis to be automatized and offer new models for quantifying and investigating the textual data.

### Existing approaches

Strategies that have been employed for the automated analysis of free texts in the social sciences can be classified into two broad categories. First, there are strategies that follow a top-down logic, which might also be referred to as deductive methods or closed-vocabulary approaches. Second, there are strategies that follow a bottom-up logic, and these are primarily data driven (i.e. inductive methods, open-vocabulary approaches).

Top-down approaches rely on existing word lists, called dictionaries, that organize certain words, parts of speech (e.g. pronouns), or other textual properties (grammatical structure, punctuation) into specific categories. They are employed in, for example, the field of sentiment analysis, which is the attempt to use NLP to recognize emotions, opinions, and attitudes toward entities in textual data [5]. Sentiment analysis is applied broadly, ranging from observations of the public’s attitudes toward political movements to movie sales predictions (see [5, 6] for various applications), and sophisticated technological approaches have been developed for these purposes (see [6] for a comprehensive survey). Nevertheless, simple keyword spotting (e.g. classifying texts on the basis of unambiguous affect words) is still popular because it is accessible and economical [7].

A specific tool that was created with a top-down approach is the LIWC [8, 9]. This software counts words according to categories that are considered to be relevant for psychological research and resulted from exhaustive preliminary studies. The authors of the software distinguish between content words that convey the content of the communication (e.g. “write,” “scientific,” “paper”) and style words that are needed to form phrases (e.g. “I,” “a,” “and”). The latter make up large parts of written and vocal speech. Tausczik and Pennebaker suggested that style words are more closely linked to people’s social and psychological worlds [8], citing studies that link, for example, pronoun use to relationship quality. The LIWC is a popular and well-established tool amongst psychologists, and its dictionary has been translated into more than 10 languages; the tool has also been praised for its user friendliness [10].

The most evident disadvantage of software that is based on predefined dictionaries is a lack of flexibility. The categories that are employed–no matter how well-validated they are within a specific context–might not cover the aspects of interest in the respective study, might not apply to the specific type of text used, or might miss important information that the researcher is not aware of when pre-specifying the categories of interest.

Bottom-up approaches avoid these problems by deriving relevant categories or properties from textual data itself instead of relying on predefined dictionaries. For example, Schwartz et al. [11] introduced the term open-vocabulary technique to describe their bottom-up analysis of Facebook messages. They analyzed words and phrases consisting of two to three consecutive words (so-called n-grams, e.g. “my children”), as well as topics derived from the texts through Latent Dirichlet Allocation (LDA). LDA is a generative topic model in which texts are assumed to share a certain number of underlying topics, which explain co-occurrences of words within texts [12]. Using this technology, Schwartz et al. found for example that one topic in Facebook status updates was *family* (“son,” “daughter,” “father,” etc.), and this topic occurred more frequently in the status updates of older users. By contrast, the topic *studies* (“classes,” “semester,” “college,” etc.) was more relevant to younger users. The study furthermore introduced the term differential language analysis (DLA) to describe how simple ordinary least squares regressions could be used to link word use with characteristics of the author of the text.

Besides topic modeling, cluster analysis is a second popular bottom-up approach. Whereas topic models attempt to identify topics that underlie the text documents (e.g. topic “family” vs. topic “work”); cluster analysis attempts to sort the text documents into meaningful categories (e.g. documents that fall into the category “family” vs. documents that fall into the category “work”). Thus, both approaches can be applied to find meaningful units in a number of text documents but differ regarding the statistical model and can lead–depending on the features of the text documents–to either similar or diverging results.

As an example from political science, Grimmer and King [13] applied not only one but *all* published cluster analysis methods to find meaningful partitions in press releases of US Senator Frank Lautenberg’s Senate Office, George W. Bush’s 2002 State of the Union address, and randomly drawn Reuters news stories. Their unique method revealed that different algorithms lead to clusterings that can be organized in a two-dimensional space. On the basis of this space of clusterings, they discovered that Lautenberg’s press releases could be organized into four clusters: *Credit Claiming*, *Advertising*, and *Position Taking*–traditionally considered to be the three basic kinds of activities that congressmen engage in [14]–and a new fourth cluster that they labeled *Partisan Taunting*.

For the implementation of bottom-up approaches, several software packages such as Leximancer [15] and SPSS Text Analytics for Surveys [16] offer social scientists the means to process textual data and run procedures such as topic detection. However, these programs are rather expensive, hinder replication, and lower transparency through the “black box” characterization that often comes with proprietary software. Noncommercial and open alternatives that offer a wide range of similar functions include the R-package tm [17] and collections of tools such as the Apache OpenNLP library [18], the Natural Language Toolkit for Python [19], and the ASV Toolbox [20].

Not all approaches to automated text analysis fall neatly into the idealized categories top-down versus bottom-up. In supervised learning, part of the data need to be labeled. For example, a certain number of texts is classified by trained human coders. In the next step, an algorithm is trained on the basis of these labeled data to infer the assigned class from the texts, and this trained classifier can then be used to classify new texts automatically. Supervised learning could be considered top-down because the relevant categories are imposed in the first step. However, it derives the relevant features for distinguishing the categories from the text itself in a bottom-up manner. Research on authorship attribution has been drawing from such methods since the 19^th^ century: A number of texts for which the author is known (labeled data) can be used to identify the features that distinguish between multiple candidate authors with the potential to identify the authorship of documents of uncertain origin. Applications range from the identification of Shakespeare plays to the verification of suicide notes, see [21] for a survey of modern authorship attribution methods.

As a potential application of supervised learning in the social sciences, Hopkins and King [22] used automated content analysis to investigate opinions about the 2008 US presidential candidates. A few hundred blog posts were hand-coded, ranging from extremely negative to extremely positive. Automated analysis then allowed the authors to estimate opinions in a large corpus of blog posts on the basis of the training data. For example, results revealed a sharp increase in negative opinions about John Kerry following his botched joke that was perceived as an insult to the troops in Iraq in October 2006 (“If you make the most of it and you study hard and you do your homework and you make an effort to be smart, you can do well. If you don't, you get stuck in Iraq”). The authors of this paper wrote an R-package to make the method they developed accessible [23].

### Challenges of the analysis of textual data

Despite readily available software solutions and the advances of NLP technologies, core features of human languages still render automated analyses difficult. By combining words, an unlimited amount of utterances can be produced, which can be–even if the combination is novel to the listener or reader–easily understood by other humans. This feature has been labeled “productivity” [24]. Productivity is even reflected in object naming tasks: Participants tend to generate variable and sometimes quite inventive answers, a phenomenon that has been described as exuberant responding [25], which can cause issues in research on speech production in standardized experiments. It seems plausible that variability in answers increases even more when proceeding from simple tasks to questions addressing a respondent’s social life, problems, interests, living conditions, and so forth. To the human recipient, this does not cause any issues in most cases. For example, one can easily see that the phrases “My wife doesn’t let me meet my friends” and “Spouse’s impact on friendships” refer to a similar problem. However, it is a major challenge for automated analyses to detect semantic similarities between two such answers that share hardly any common substrings.

Data pre-processing can tackle variability in human languages to a certain extent. For example, we might be able to reduce “friends” and “friendships” in the two phrases to a common word stem to gain the insight that both strings have something to do with “friend.” In an even more sophisticated approach, we might be able to automatically look up spouse and wife in a dictionary–in this case, more specifically in a WordNet that groups words and labels their semantic relations–and figure out that they refer to similar concepts. Another source of variability than can be reduced through NLP is flawed data input. Respondents might write down their answers themselves or dictate them to the interviewer. In both cases, spell-checking can become a necessary data pre-processing step because pairs of non-matching strings such as “mispelling” and “misspelling” cannot be mapped onto each other. Decreasing homogeneity through pre-processing with these kinds of steps improves simple analyses such as word counts because words carrying similar or identical semantics can be identified as such even when the strings are not strictly identical.

However, this “normalization” might also lead to a loss in the richness of the original answers and their individual style. There is no convention that governs the extent to which free texts should be altered, and the steps of pre-processing largely depend on the aim of the analysis. For example, so-called stop words such as “the” and “and,” which are typically the most frequent words in any language, are often dropped before the automated classification of textual data because they are not supposed to carry any significant content [26]. Yet these words strongly overlap with the concept of style words that are considered of special importance from the psychological perspective of the LIWC [8] and have been found to be the best features for discriminating between authors in the context of authorship attribution [27]. A one-size-fits-all solution for automated text analysis is currently neither available nor attainable: A suitable analysis strategy always depends on the properties of the text and the aim of the analysis.

### Aim of this paper

The aim of this study is to demonstrate pragmatic analytical strategies for free texts generated by open-ended questions as such questions are frequently included in large social science surveys. We offer guidelines on how to make use of such data in the context of quantitative social science research. The analyses follow a data-driven approach so that the results do not rely on pre-defined categories of interest. They draw on statistical procedures that are well-known in the social sciences (e.g. regression models such as OLS and logit models) and existing tools used by the NLP community. Grammar and word order are ignored in all analyses of textual data, following a so-called bag-of-words approach. Instead of pinning down certain software solutions, we will emphasize the concepts and the rationale behind the steps used in the analysis of textual data.

**Fig 1:**
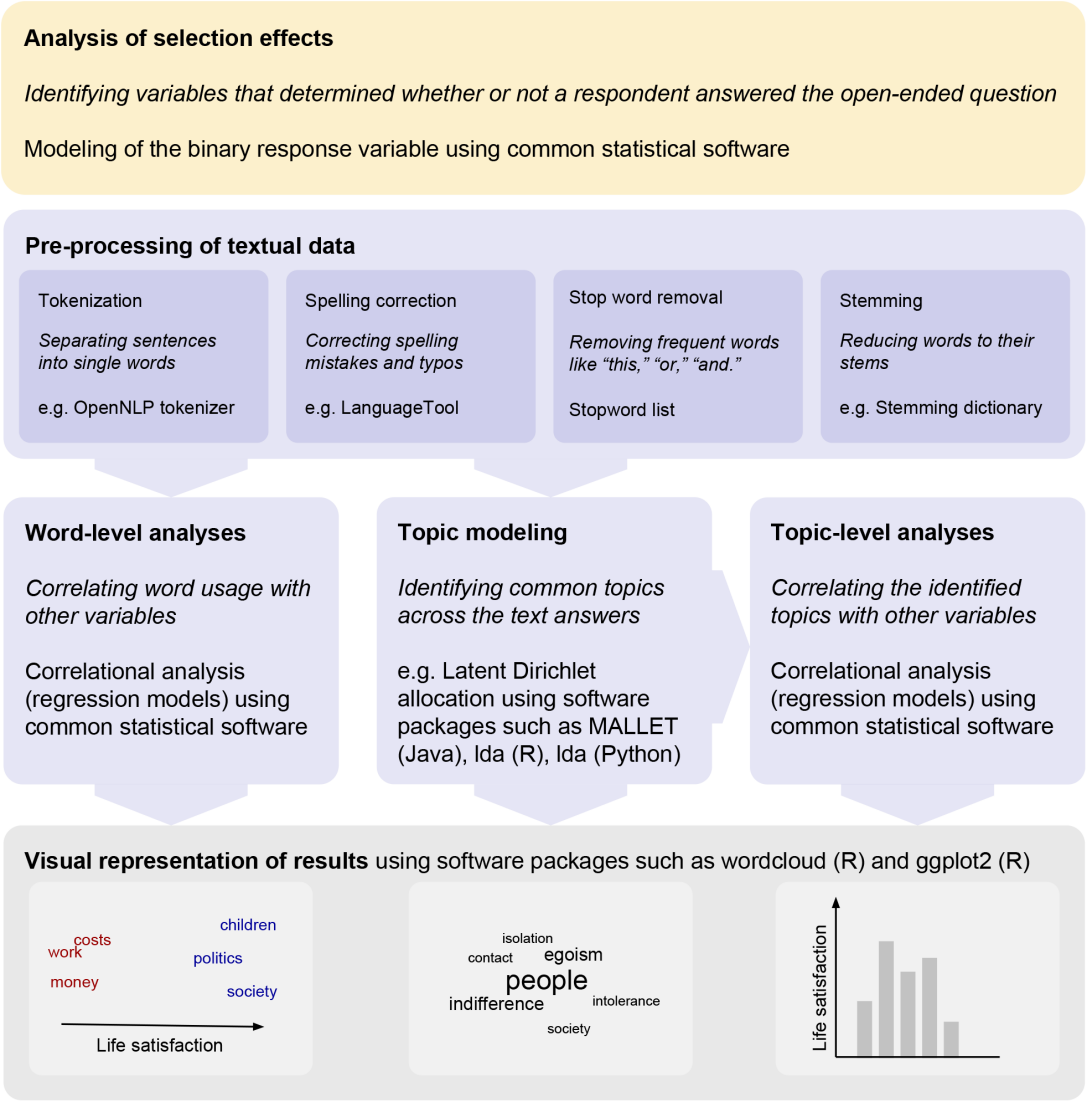
Overview of the steps of automated text analysis.

To illustrate this process, we analyzed textual answers to the question “What else are you worried about?” from a large-scale longitudinal study. The open-ended question followed a block of close-ended questions about worries. The free texts were typically short, ranging from single words to brief lists or simple sentences. Fig 1 presents an overview of the steps used in data pre-processing and the analyses that were applied in this study. Analysis code can be accessed via the Open Science Framework (https://osf.io/aj3bn/). We attempted to answer three broad research questions that are of general interest when analyzing textual data from survey studies:

1. Which respondents make use of the open-ended questions?
2. Which topics can be found in the answers to these questions?
3. How are free texts linked to respondents’ characteristics?

These questions can be rephrased more specifically given the data at hand: Who reports worries in a textual format? What are respondents worried about? And who worries about what?

## Method

### Data

The data came from the Socio-Economic Panel (SOEP), a representative prospective multi-cohort study of people living in private households in Germany [28]. The SOEP, a research infrastructure unit of the Leibniz Association (http://www.leibniz-soep.de), is located at the German Institute of Economic Research (DIW Berlin), and the data are collected by the commercial fieldwork organization TNS Infratest Sozialforschung (Munich). SOEP data have been gathered annually since 1984, and the sample has been refreshed several times to ensure representativity. In this study, we used data collected from the years 2000 to 2011, yielding a total of 261,894 records (i.e. completed questionnaires) from 44,506 individuals. On average, there were 21,800 records per year, with a minimum of 19,127 in 2010 and a maximum of 24,576 in 2000.

### Variables

#### Mode

The SOEP employs different modes of interviewing; mainly the CAPI (Computer Assisted Personal Interview, 30.25% of all of our observations), oral interview (25.17%), written questionnaire in the presence of an interviewer (24.85%), and written questionnaire sent via mail (12.67%). The interviewing mode was coded as either (1) oral (oral interview, CAPI, phone interview), (2) written (questionnaire with or without the presence of an interviewer, written, sent via mail), or (3) mixed/other (with interviewer assistance, oral and written, proxy).

#### Gender and age

Respondents’ gender was assessed via self-report and coded 0 if female and 1 if male. Age was derived from respondents’ year of birth and the survey year. To disentangle between-subjects age effects from within-subjects age effects, we generated two variables: The mean age of the person across all of his or her records to capture between-subjects variability and the difference between a person’s current age and his or her mean age to capture within-subjects variability [29].

#### Region

The variable “sample region” was used to differentiate between individuals living in East and West Germany. The variable was entered into the analyses as a binary variable, representing the region in which the respondent had spent the majority of the survey years.

#### Level of education

Information on the highest level of education was available for multiple years. The most recent information was used as the indicator of the participant’s level of education. In Germany, children are separated into one of multiple tracks of secondary education after elementary school, which leads to a number of different school leaving qualifications. In the multilevel analysis of selection effects, education was included as a qualitative variable with five levels: (1) “no degree,” (2) lower secondary education (final examination after Grade 9, *Hauptschulabschluss*), (3) middle secondary education (final examination after Grade 10, *Mittlere Reife/Realschulabschluss*), (4) intermediate higher secondary education (final examination after Grades 11 or 12; entitled to study at a University of Applied Sciences, *Fachhochschulreife*), (5) higher secondary education (final examination after Grades 12 or 13; entitled to study at all types of universities, *Abitur*), “no degree yet” and “other degree.” For correlational analyses, individuals with “no degree yet” or “other degree” were dropped, and the variable was assumed to be ordinal, with level of education increasing from (1) to (5).

#### Immigration background

Respondents’ history of migration was originally reported in four categories: “no migration background,” “direct migration background” (born outside of Germany), “indirect migration background” (born in Germany, at least one parent born outside of Germany), and “migration background, not further differentiated” if there was no information on whether the respondent was born in Germany or not. The last category accounted for only 0.06% of the observations, which were recoded into “indirect migration background” to simplify the analyses.

#### Personality

Personality was assessed in 2005 and 2009 with a brief personality questionnaire (BFI-S [30]). The BFI-S consists of 15 items answered on a 7-point scale to capture the five broad personality dimensions extraversion, emotional stability, agreeableness, conscientiousness, and openness to experience. Scale reliability ranged from α = .50 (agreeableness) to α = .68 (openness to experience). Due to the brevity of the BFI-S, scales were computed only when all 15 items had been answered; in 0.5% of the observations, respondents had answered only parts of the BFI-S. Thus, we did not calculate personality scores. If self-reported personality was available for both points of measurement, we averaged the scores.

#### Life satisfaction

Life satisfaction was assessed every year with a single item on an 11-point scale. As described for age, within-subjects centering was used to derive two variables, the mean level of a person’s life satisfaction (ignoring missing records) and the deviation from that mean level in a specific year.

#### Closed-ended worry items

Respondents answered a number of closed-ended questions regarding their worries about various subjects on a 3-point scale (“not worried at all,” “somewhat worried,” and “very worried”). Nine items were included in all surveys from 2000 to 2011 (worries about the general economic situation, personal financial situation, personal health, job security, protection of the environment, peace, development of criminality in Germany, immigration to Germany, and hostility toward foreigners in Germany). Additional items addressing current issues were included intermittently over the course of the study (e.g. worries about the introduction of the Euro, global terrorism, the stability of the financial markets, and the security of nuclear power plants).

We averaged eight of the nine items that were asked on every survey to form a score of reported worries that was comparable across survey waves. The item regarding worries about job security was excluded because it applied only to the subset of individuals who were employed. Scores were computed if at least seven of the eight items were answered but not for 0.47% of observations in which one to six items were answered or 1% of observations in which none of the eight worry items were answered. The resulting scale had an acceptable reliability coefficient (α = .73, pooled across all waves). Again, within-subjects centering was used to derive two variables, a person’s mean level of reported worries and the person’s level of deviation in a specific year.

### Textual data

After receiving the block of worry items, participants were asked whether they had any other worries. Answers to this open-ended question (written by participants or transcribed by interviewers) were cleaned and prepared for analysis in Java. The response language was German, which has more inflections than English (e.g. words are modified according to case and gender) and uses more suffixes and therefore imposes some particular challenges.

Texts were set to lowercase because capitalization had been used inconsistently by respondents and interviewers, and information about part of speech (which is related to capitalization in German) was not considered in further analyses. Texts were then tokenized (e.g. broken down into single words) by applying the OpenNLP tokenizer [18]. Character encoding varied between different waves of the panel, and thus, encoding needed to be unified to prevent special characters from being misrepresented. Furthermore, respondents and interviewers used abbreviations because of the space limitations on the questionnaire. We thus assembled an ad hoc list of common abbreviations (e.g. “soz.” to “sozial,” *social*; “dtl” to “Deutschland,” *Germany*; see S1 List) and the most conspicuous spelling errors (e.g. “standart” to “standard,” *standard*) and replaced the strings accordingly.

In the next step, stop words were removed from the data. Note that there is no such thing as a universal or official stop word list. In this study, we used a German stop word list that was based on the Leipzig Corpora Collection [31]. Finally, words were reduced to their word stem (e.g. “politischen,” *political*, and “politiker,” *politicians*, to “polit”; “kind,” *child*, and “kinder,” *children*, to “kind”) by applying the German Snowball stemmer list [32, 33] but then re-expanded (e.g. “polit” to “politik,” *politics*, “kind” to “kinder,” *children*) by applying a custom list to improve readability, see S2 List.

**Fig 2:**
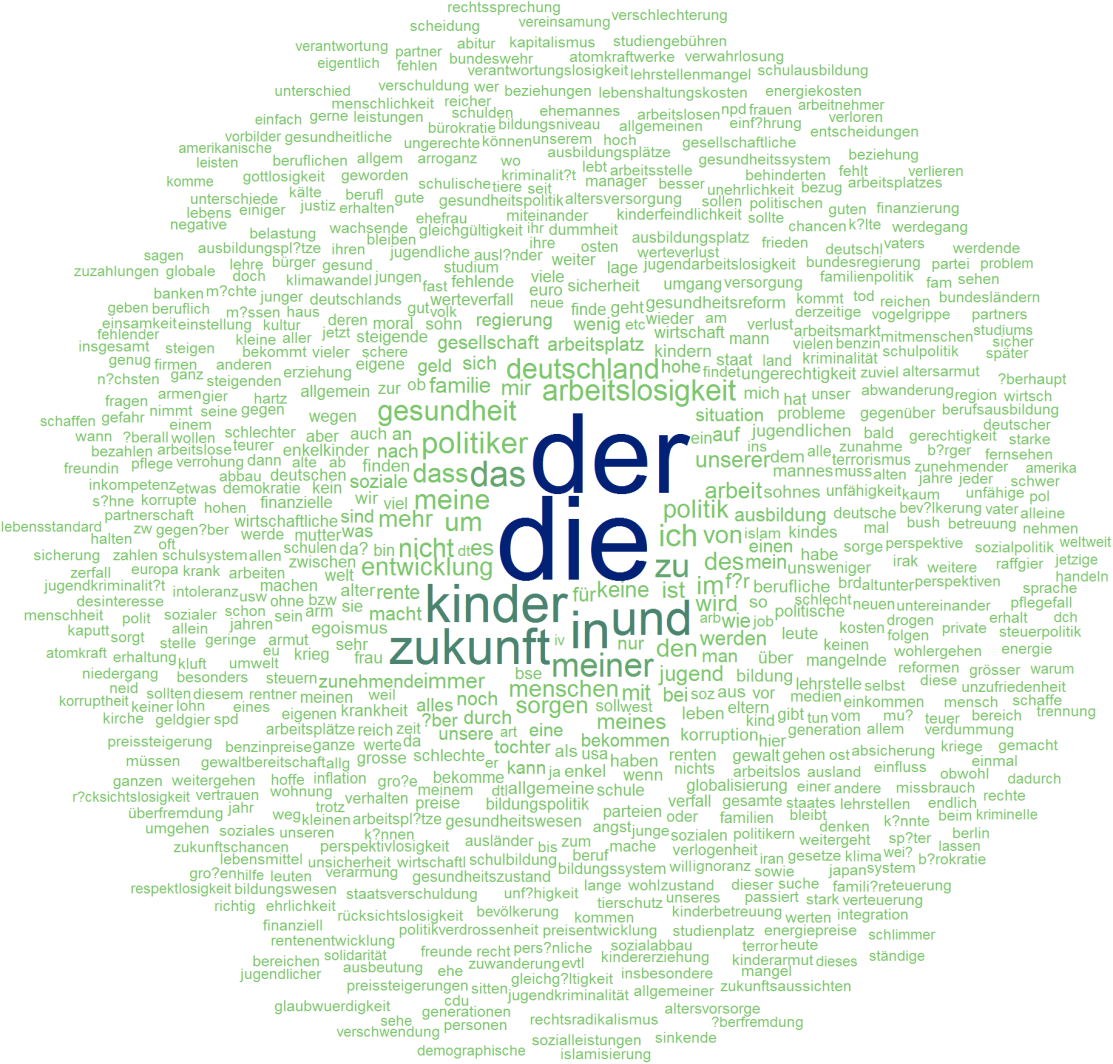
Word cloud of “raw” texts, tokenized but not otherwise processed.

**Fig 3:**
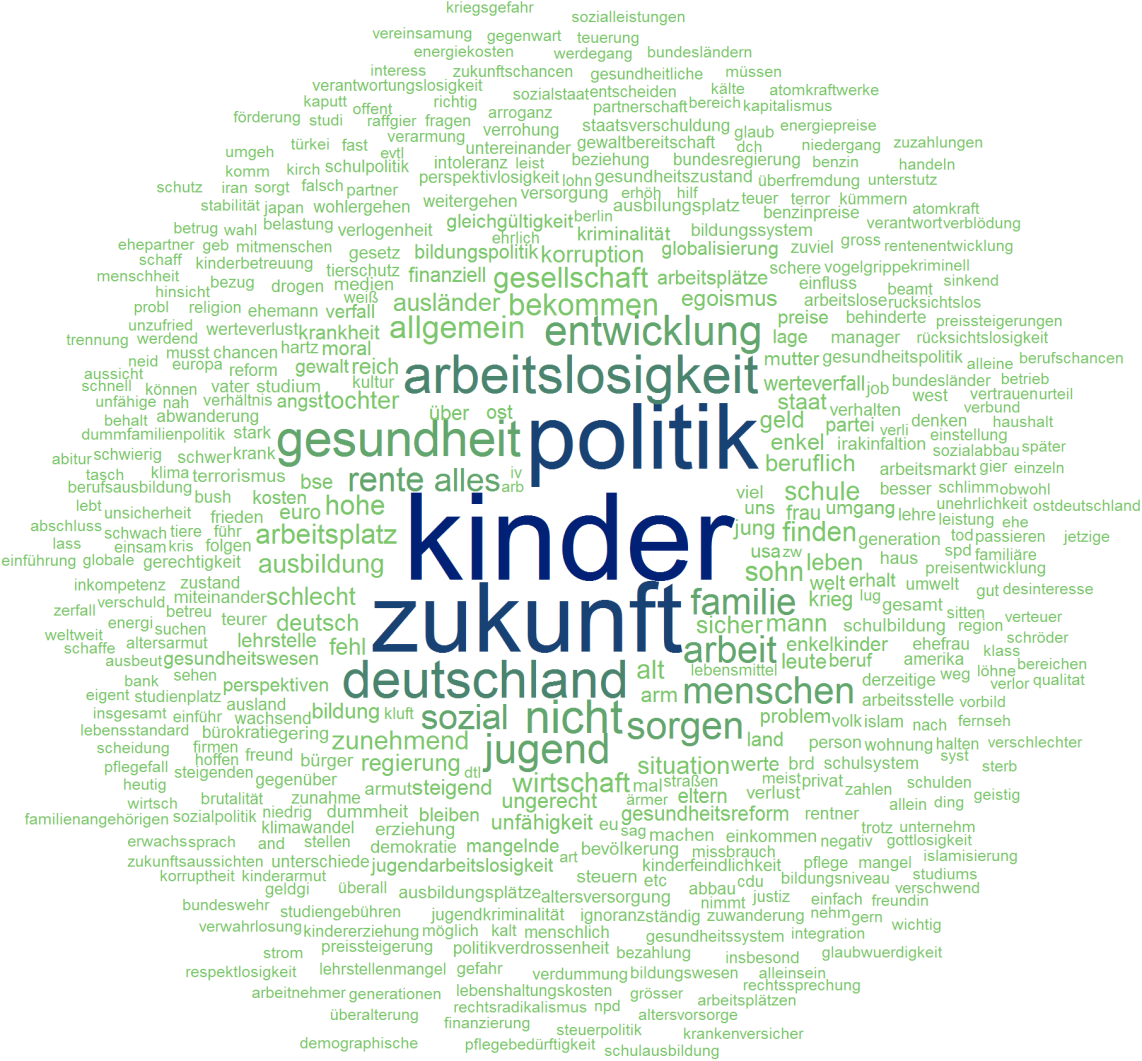
Word cloud of free texts after data pre-processing.

Fig 2 shows a word cloud of the tokenized but not further edited texts and visually represents the “raw” textual data. Note that two definite articles, “der” and “die,” are the most frequent words, followed by several other stop words such as “in” (*in*) and “und” (*and*). Furthermore, multiple wrongly encoded special characters displayed as question marks within words are visible. Fig 3 shows a word cloud that represents the prepared data that were used in further analyses. Note that at this point, meaningful words dominated the cloud, i.e. “kinder” (*children*), “zukunft” (*future*), and “politik” (*politics*). The contrast between these two word clouds illustrates how the pre-processing steps eliminated irrelevant words and flawed strings.

Words were translated into English just before the visual representations were created so that the translation process had no impact on the results of any analysis. We used a manually compiled ad-hoc list (see S3 List), which was compiled with the help of several online German-English dictionaries. The translation is not a one-to-one mapping and can thus lead to a number of peculiarities. For example, German compound words (e.g. “lehrstelle,” apprenticeship position) translate into multiple words that would have been split by tokenization if the analyses had been run on English textual data, and in some cases, multiple German words were mapped onto the same English word (e.g. “lehrstelle” and “ausbildungsplatz,” both apprenticeship position). In addition, the different levels of inflection and other specifics of the translation from German to English rendered the process a bit fuzzy overall. However, we considered this step to be necessary to illustrate the results for a broader audience.

### Latent Dirichlet Allocation (LDA)

Latent Dirichlet Allocation is a general probabilistic model that can be used for collections of texts [12]. Every document (i.e. textual answer) is modeled as a combination of topics, and topics are modeled as distributions of words. LDA requires a number of parameters to be predefined, that is, *α*, the Dirichlet prior on the per-document topic distributions; *β*, the Dirichlet prior on the per-topic word distribution; and the number of topics to be modeled. The Dirichlet priors influence whether a document may contain more or fewer topics and whether topics tend to be distinct or contain a mixture of most words. Typical quantitative measures of model fit that help to determine the adequate number of topics are based on likelihood. However, human ratings of the coherence of the derived topics do not necessarily line up with these statistics [34]. In this study, multiple analyses were run, modeling one up to 100 topics. Priors were fixed to *α* = 50/*No*. *of topics* and *β* = 0.1 as suggested by Griffiths and Steyvers [35].

### Correlational analyses

Schwartz et al. [11] suggested correlational analyses to identify words that potentially distinguish between respondents with different features (e.g. words that indicate older age, low extraversion, etc.). Their model is a simple ordinary least squares regression with the desired dependent variable (e.g. life satisfaction) and a binary variable that indicates whether a specific word (e.g. “job”) was used by the respondent as the independent variable. Furthermore, age and gender are also included as independent variables to control for potential confounding. We followed their approach but chose the statistical model according to the level of measurement of the dependent variable: ordinary least squares regressions for the continuous dependent variables age, life satisfaction, extraversion, emotional stability, agreeableness, conscientiousness, and openness to experience; logit regressions for the binary dependent variables gender and sample region; and an ordered logistic regression for the ordinal dependent variable education. Two control variables, gender and age, were included unless the respective variable was the dependent variable in the analysis. The outcome of interest was the standardized regression coefficient of the word use variable or the semi-standardized regression coefficient in the case of logistic regression.

We ran analyses for all 243 words that appeared at least 50 times in the cleaned textual data. All analyses were run twice: one time on the subsample of people who answered the open-ended question and a second time on the full sample, thus including individuals who did not answer (coded as 0 for the use of every word that was analyzed).

Furthermore, we ran correlational analyses with topic occurrence as modeled by the LDA as an independent variable. LDA returns a distribution of topics for every textual answer; the probabilities of topic occurrence sum to 1. To simplify the analyses, occurrence of a specific topic within a text was dichotomized on the basis of the probability of topic occurrence. Values equal to or larger than .3 were coded as topic occurrence (1) and values below .3 as no topic occurrence (0). Preserving the original outcome variables (the probabilities for each topic within each text) led to virtually the same results in all of the following analyses. However, the results were easier to interpret when they were based on dichotomous topic occurrence variables instead of continuous topic occurrence probabilities. Apart from the different nature of the independent variable (topic occurrence instead of word occurrence), the analyses of topics paralleled the correlational analyses of single words with regard to the statistical model and control variables.

## Results

### Selection effects

Which respondents made use of the open-ended question, or more specifically: Which variables determined whether a respondent answered or ignored the question? We ran multilevel binary logistic regressions predicting the binary outcome answer/no answer to investigate selection effects (see Table 1). All analyses of selection effects were run on the sample of 221,881 records of 25,952 respondents in which all variables included in the final model were non-missing.

**Table 1:** Results of binary logistic multilevel regressions predicting responses to the open-ended question, including 222,165 records from 25,978 individuals.

		Model 1		Model 2		Model 3	
Trait (unit of change)		Odds Ratio	*p*	Odds Ratio	*p*	Odds Ratio	*p*
*Gender*–female							
	Male	0.87	< .001	0.87	< .001	0.97	.420
*Age* (1 year)							
	Between	1.01	< .001	1.01	< .001	1.02	< .001
	Within	0.98	< .001	0.98	< .001	0.98	< .001
*Sample Region*–West							
	East	1.36	< .001	1.23	< .001	1.24	< .001
*Education*–Lower secondary							
	Middle secondary	1.43	< .001	1.50	< .001	1.39	< .001
	Intermediate higher secondary	1.99	< .001	2.15	< .001	1.89	< .001
	Higher secondary	2.24	< .001	2.52	< .001	2.20	< .001
	Other	1.32	.001	1.35	< .001	1.35	.001
	None	0.96	.760	0.90	.430	1.01	.959
	Not yet	1.71	< .001	1.89	< .001	1.77	< .001
*Migration background—*none							
	Direct	0.79	.001	0.77	< .001	0.78	< .001
	Indirect	1.13	.060	1.12	.070	1.11	.090
*Survey mode*–verbal							
	Written	0.90	< .001	0.86	< .001	0.86	< .001
	Mixed	0.86	.001	0.83	< .001	0.82	< .001
*Life Satisfaction* (1 point)						
	Between			0.85	< .001	0.84	< .001
	Within			0.91	< .001	0.91	< .001
*Big Five* (1 SD)							
	Emotional Stability					0.88	< .001
	Extraversion					1.07	< .001
	Agreeableness					0.98	.286
	Conscientiousness					1.01	.585
	Openness					1.33	< .001
*Model evaluation*		*Χ*^2^	*p*	*Χ*^2^	*p*	*Χ*^2^	*p*
	Fit (against Null model)	670.82	< .001	1083.72	< .001	1613.47	< .001
	Comparison with Preceding Model			412.90	< .001	529.75	< .001

The first model included basic demographic variables (gender, age, sample region, education) and the mode of data assessment (survey mode). Men were less likely to answer the question than women (*OR* = 0.87, *p* < .001). Regarding age, the between- and within-subjects effects showed different trends. Although older respondents were more likely to answer than younger respondents (*OR* = 1.01 per year, *p* < .001), an individual became less likely to answer over time (*OR* = 0.98 per year, *p* < .001). Respondents from East Germany had remarkably higher odds of answering the questions (*OR* = 1.36, p < .001). An overall test revealed that level of education had a significant effect on their answering behavior, Χ^2^(6) = 335.45, *p* < .001, and the pattern of Odds Ratios relative to the comparison group with the lowest secondary school leaving qualification showed that respondents with higher levels of education were more likely to answer. Individuals who had immigrated to Germany were less likely to answer the question (*OR* = 0.79, *p* < .001), yet the children of immigrants were–if at all–slightly more likely to answer the question (*OR* = 1.13, *p* = .060). Last, answering behavior varied with survey mode: Respondents were more likely to answer the question in a verbal interview than on a written questionnaire (*OR* = 0.90, *p* < .001) or in a mixed survey mode (*OR* = 0.86, *p* = .001)

The second model additionally incorporated life satisfaction to test whether this subjective indicator predicted answering behavior above and beyond the objective variables entered in the first model. Life satisfaction had effects on answering behavior beyond the variables included in the first model, *Χ*^2^(2) = 412.90, *p* < .001. Both the between- and within-subjects effects were significant. Respondents who were on average more satisfied with their lives than other respondents were less likely to provide an answer to the open-ended question about worries (*OR* = 0.85 per each point of life satisfaction, *p* < .001); moreover, respondents became less likely to answer the question when they became more satisfied over the years (*OR* = 0.91 per each point of life satisfaction, *p* < .001).

Last, we tested whether personality variables that are not directly linked to worries additionally influenced individuals’ answering behavior. The third model added the Big Five personality traits extraversion, emotional stability, agreeableness, conscientiousness, and openness to experience. Including the personality traits significantly improved the model over the previous version (*Χ*^2^(5) = 529.75, *p* < .001). Most remarkably, individuals who had reported higher levels of openness to experience were more likely to provide a textual answer (*OR* = 1.33 per SD, *p* < .001). Furthermore, individuals who were more emotionally stable were less likely to answer (*OR* = 0.88 per SD, *p* < .001). A comparatively small effect was found for extraversion: More extraverted respondents were slightly more likely to answer (*OR* = 1.07 per SD, *p* < .001).

#### Relationship between open- and closed-ended questions

In an additional analysis, we tested whether a higher worry score from the closed-ended questions was associated with answering the open-ended question. In the analysis including only the worry score as a predictor, both the between- and within-subjects effects were statistically significant. Respondents who answered affirmatively to more worry items were also more likely to answer the open-ended question about worries: A difference of 1 point (e.g. answering all worry items with “slight worries” vs. answering all items with “no worries”) doubled the odds of answering the open-ended question (*OR* = 2.01, *p* < .001). Likewise, an individual who ticked more worry items than he or she had ticked in other years was also more likely to answer the open-ended question in the respective “worrisome” year (*OR* = 1.86, *p* < .001). Including socio-demographic variables, life satisfaction, and the Big Five personality traits (i.e. all variables included in the third model described above) decreased the between-subjects effect of the worry score (*OR* = 1.57) but left the within-subjects effect nearly unchanged (*OR* = 1.83). Both effects retained their significance (*p*s < .001). Thus, reports of worries across different answer formats shared common variance between-subjects as well as within-subjects, a finding that persisted after accounting for several socio-demographic and personality variables.

### Topic detection

#### Topic model

**Fig 4:**
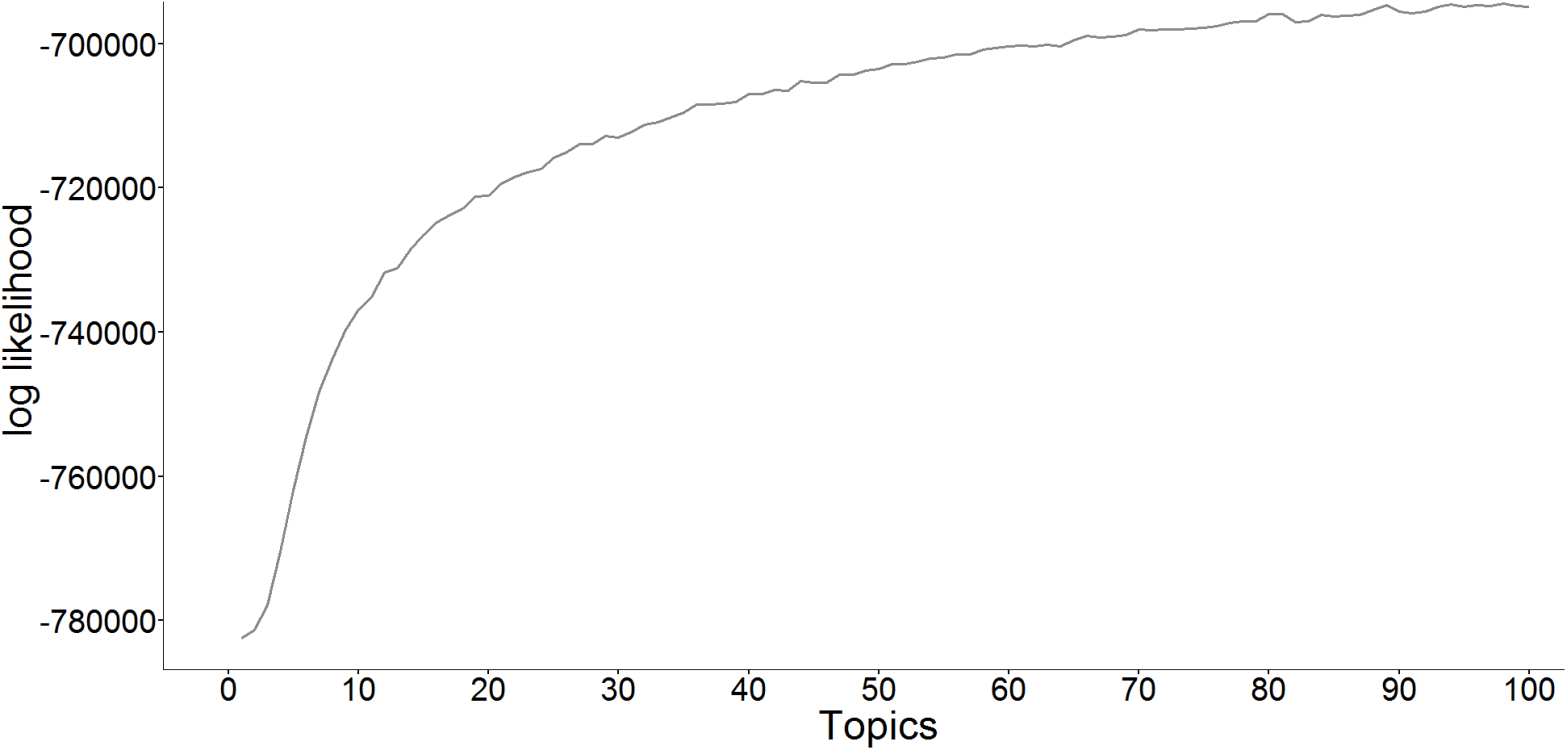
Log likelihood of LDA models, depending on the number of topics chosen.

What else were the people worried about? To answer this question, we first had to decide how many topics were represented in our model. Fig 4 displays the log likelihood of the data given the topic model: From left to right, the model fit improved, but parsimoniousness decreased as the number of topics increased. On the basis of these numbers, we estimated that at least 10 topics were necessary to cover the steepest increase in model fit. We then individually examined the per-topic word distributions and stopped at the model consisting of 15 topics, which yielded the desired degree of abstraction. Jacobi et al. [36] compared the decision about the number of topics to the decision about the number of factors in a factor analysis: The goal is to reduce the number of dimensions effectively (i.e. to find a parsimonious model) but also to lose as little information as possible (i.e. to achieve high model fit). A more objective approach for determining the adequate number of topics might be desirable, but, as mentioned before, statistical approaches do not necessarily lead to results that are aligned with human judgments of coherence [34].

**Fig 5:**
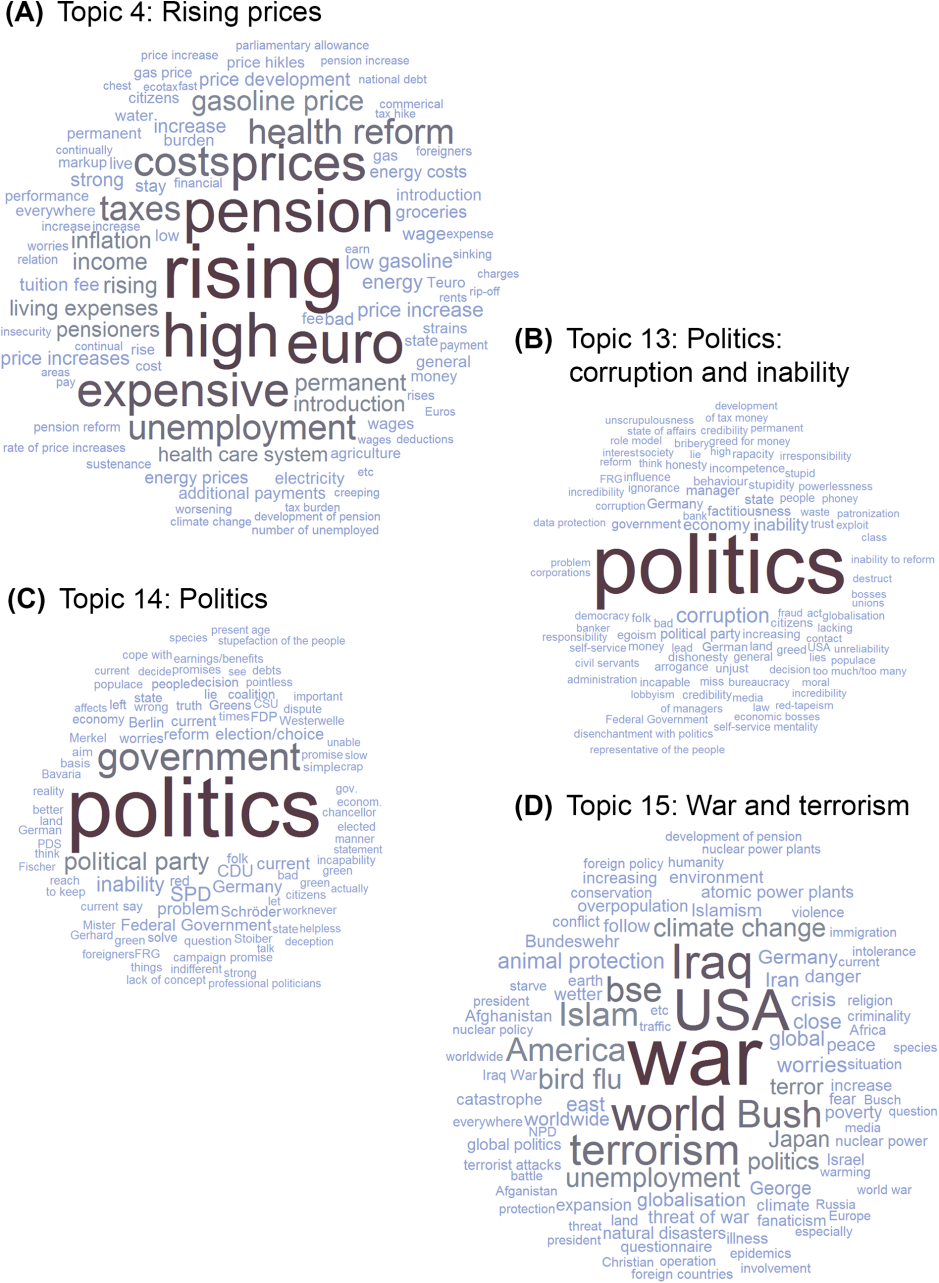
Four of the 15 topics derived through LDA topic modeling.

Fig 5 shows a selection of the resulting topics represented as word clouds. The size of the word represents the probability of a word appearing within the respective topic (i.e. the per-topic word distribution). We chose to present Topics 13 and 14 because they illustrate how topics that are centered around the same word (politics) can capture very different sentiments; we will later discuss Topics 4 and 15 with respect to their trends over time. Word clouds for all 15 topics can be found on the OSF project page (https://osf.io/aj3bn/). Topic labels were derived from the most frequently occurring words within a topic. Table 2 contains the 10 most relevant terms for each topic and the proportion of texts in which the topic occurred, according to the final LDA model.

**Table 2:** Labels of the topics derived via LDA topic modeling, proportion of texts in which the topic occurred, and the most relevant terms.

Topic	Topic name	Occurrence	Most relevant terms
1	Future of children	19.8%	children, future, occupational, youth, development, worries, grandchild, apprenticeship, son, family
2	Children, youth, school	6.0%	children, youth, school, education, apprenticeship, violence, bad, upbringing, Germany, educational policy
3	Health of family	12.4%	Health, family, man/husband, worries, children, woman/wife, son, parents, mother, daughter
4	Rising prices	6.1%	rising, high, pension, Euro, prices, expensive, costs, unemployment, health reform, taxes
5	Rich and poor	4.0%	social, rich, poor, unjust, Germany, divide, reduction, justice, peace, gap
6	Foreigners in Germany	4.0%	foreigners, Germany, German, politics, land, criminality, law, immigration, judiciary, Hartz
7	Unemployment	6.8%	Unemployment, high, youth, young, people, Germany, east, people, jobs, emigration
8	Finding employment	6.8%	work, find, receive, job, children, apprenticeship position, apprenticeship place, son, apprenticeship, studies
9	Pension and financial security	6.5%	pension, money, live, old, work, state, receive, secure, expensive, foreigners
10	Development of Germany	11.6%	Germany, development, politics, general, unemployment, economy, situation, state, social, educational policy
11	Interpersonal dealings	7.8%	people, egoism, society, increasing, indifference, contact, general, social, decline in values, together
12	Moral decay	4.5%	society, moral, values, decay, politics, loss, media, decline in values, people, general
13	Politics: corruption and inability	9.6%	politics, corruption, inability, economy, political party, factitiousness, state, manager, Germany, government
14	Politics	3.9%	Politics, government, political party, inability, SPD, problem, Germany, CDU, current, election
15	War and terrorism	6.8%	war, USA, world, Iraq, terrorism, Bush, BSE, Islam, America, bird flu

#### Topics over time

**Fig 6:**
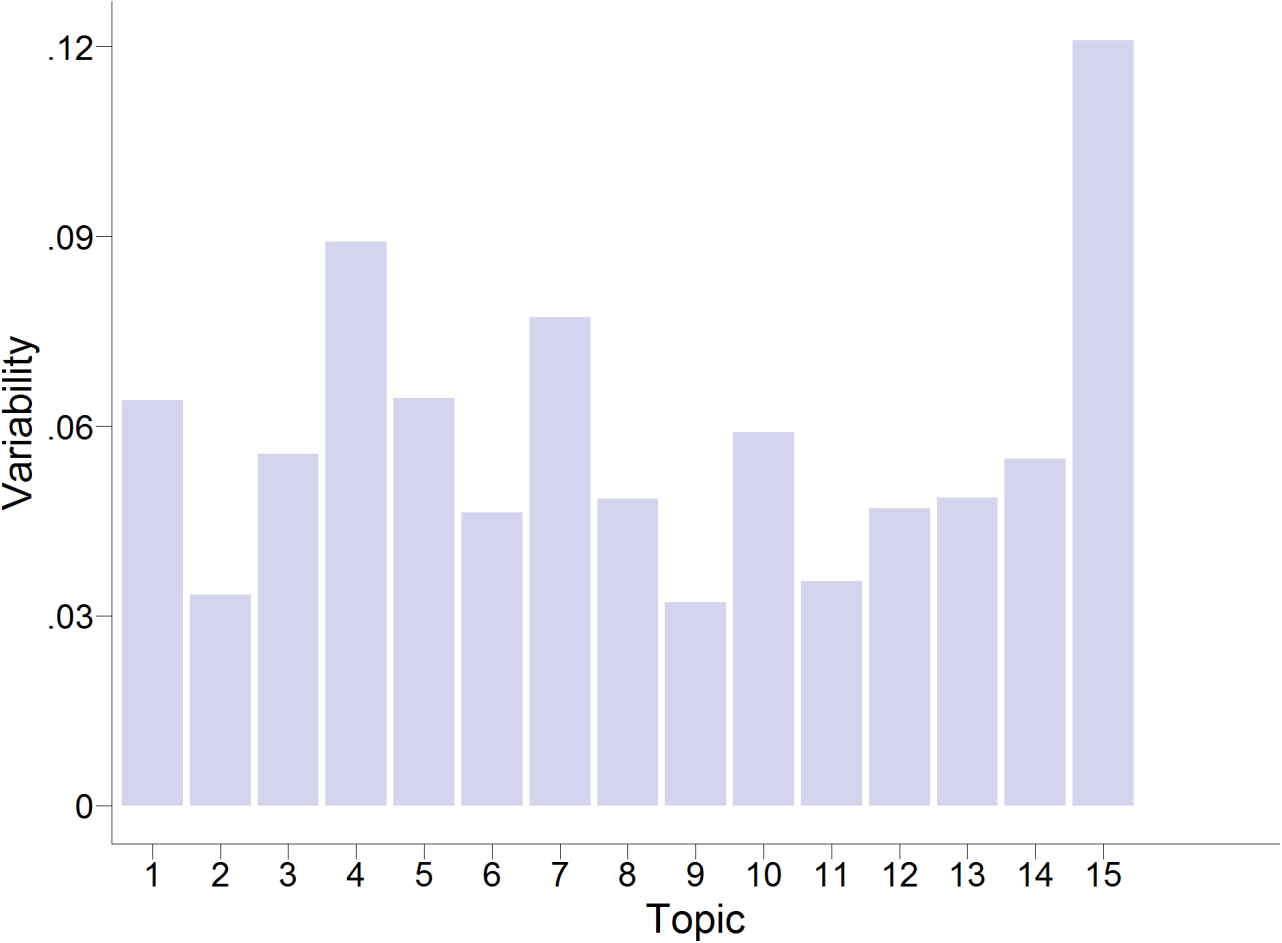
Variabilities (Coffey-Feingold-Bromberg measure) of the occurrence of topics across the years.

The occurrence of the derived topics changed over time. We calculated the measure of variability among proportions proposed by Coffey et al. [37]–the so-called Coffey-Feingold-Bromberg (CFB) measure [38]–to identify which topics fluctuated most strongly across the waves. A CFB of 0 indicates that there is no variability across the set of proportions (i.e. equal distribution), whereas a CFB of 1 indicates the maximum variability possible given the mean proportion. The CFB across survey years for each topic is displayed in Fig 6.

**Fig 7:**
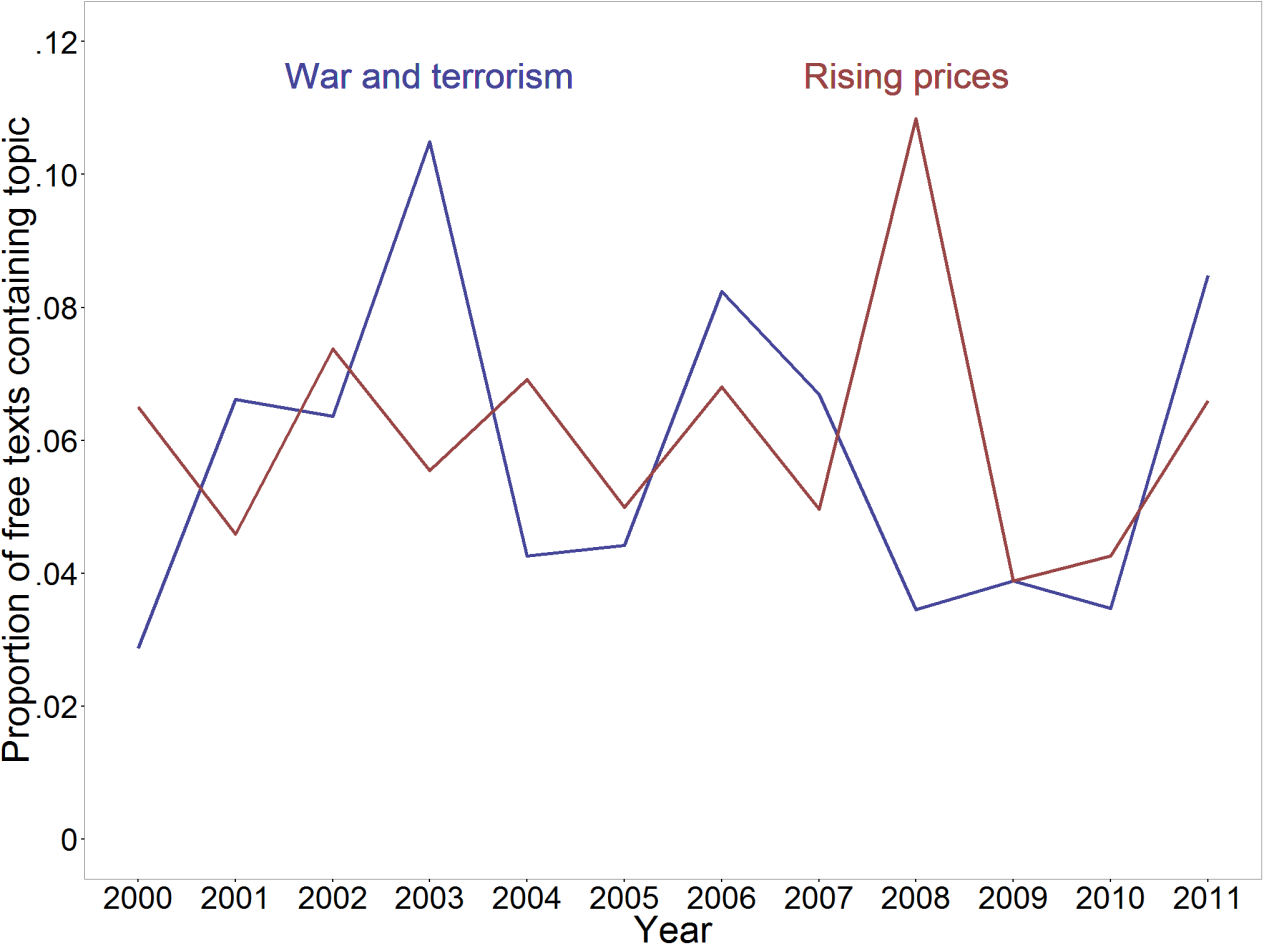
Time course of the two topics with the highest variabilities across survey years, Topic 15 (War and terrorism) and Topic 4 (Rising prices).

We identified the two topics with the highest CFB, Topic 15 (“War and terrorism,” *CFB* = .121) and Topic 10 (“Rising prices,” *CFB* = .089) and plotted their occurrence over time (see Fig 7). “War and terrorism” apparently peaked three times: in 2003 (coinciding with the onset of the Iraq War), in 2006 (coinciding with e.g. the Lebanon War), and again at the end of the interval we investigated, in 2011, when the Syrian Civil War started. Worries about “Rising prices” peaked in 2008, coinciding with the so-called Great Recession that followed the 2007 Financial Crisis.

#### Topics and closed-ended worry items

**Fig 8:**
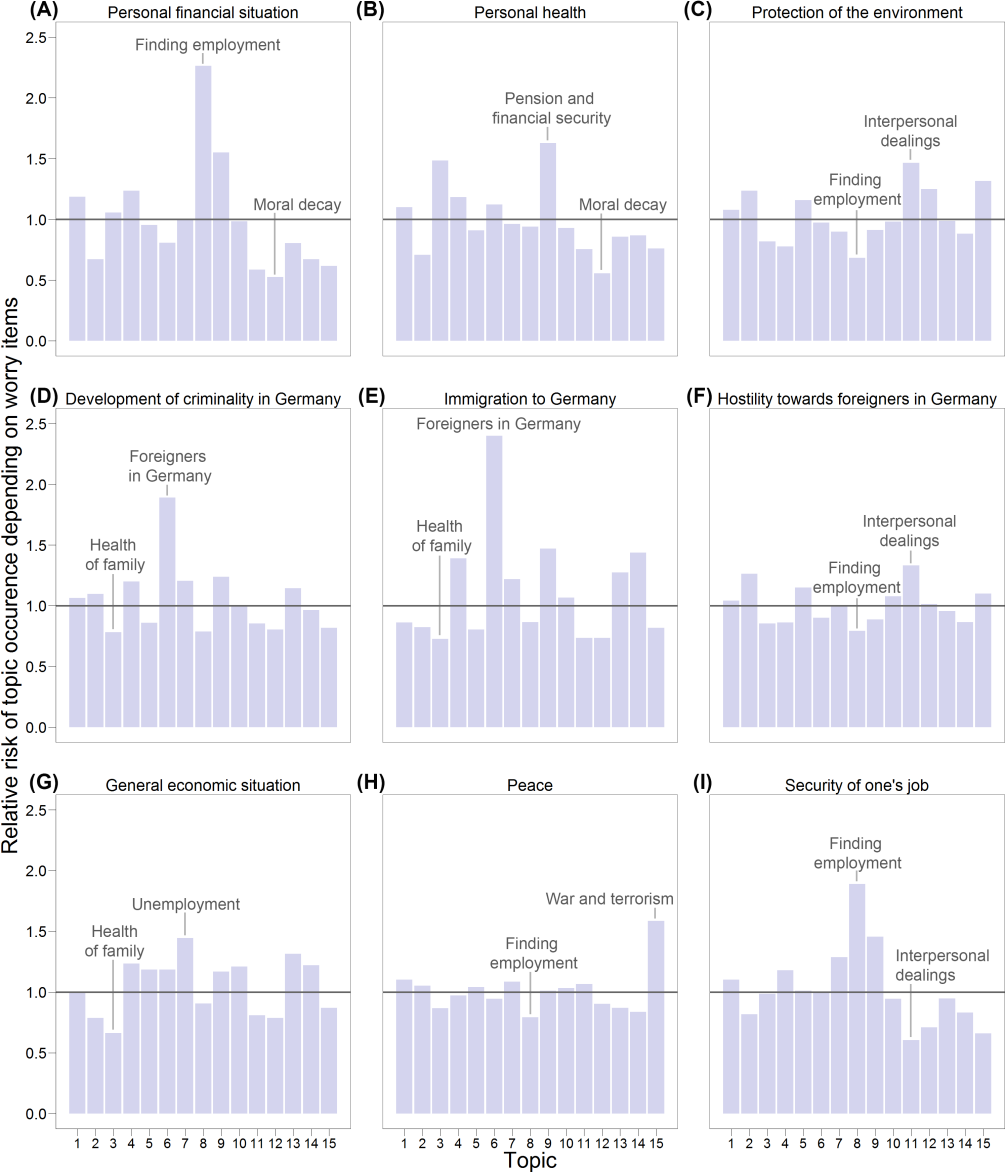
Relationships between reports of worries in closed-ended questions regarding various subjects (Panels A to I) and topic occurrence in free texts (Topics 1 to 15). Topics with the highest and lowest relative risk are labeled for each item.

How were worry topics that were reported as free text related to worries that were reported in closed-ended questions? Considering that both the closed-ended items and the open-ended question asked for worries, one would expect a certain overlap between worry topics and worry items. To analyze this, we calculated the relative risk of topic occurrence in the textual answer by comparing people who answered a closed-ended question as being “very worried” to people who reported being only “somewhat worried” or “not worried at all” about that subject. The analyses included only respondents who answered the open-ended question. Fig 8 shows the results. In many cases, the worry items were most strongly associated with similar or obviously related worry topics. For example, individuals who reported being very worried about their financial situation were 2.26 times more likely to write about the topic “Finding employment” in their textual answer. Similarly, respondents who were very worried about immigration to Germany were 2.40 times more likely to two write about the topic “Foreigners in Germany,” and respondents who were very worried about peace were 1.58 times more likely to write about the topic “War and terrorism.”

#### The influence of parameter values

In addition to testing the topic model we reported above, we ran a number of alternative specifications to probe the influence of the Dirichlet priors and the number of topics. Assuming only two topics led to a very robust solution that was hardly influenced by the choice of the Dirichlet priors: One topic most visibly centered around *future* and *children*, and a second topic centered around *politics*. Increasing the number of topics to 100 still yielded multiple topics with high face validity, such as a topic centered specifically around the *introduction* of the *euro* and the subsequent *currency devaluation* (summarized by the portmanteau “Teuro,” a contraction of “teuer,” *expensive*, and *euro*). However, many of the topics also seemed to capture idiosyncrasies of the respondents and the survey years, which were not of interest in the present study. For example, one topic was defined by various epidemics and maladies in combination with the words *Jesus* and *Christ*. The model with 100 topics also seemed most sensitive to changes in the Dirichlet priors.

When we held the number of topics constant at 15, the choice of different Dirichlet priors had a visible but weak influence on the resulting topics. As an arbitrary example, changing *α* from 3.33 to 0.01 and *β* from 0.01 to 0.80 resulted in one rather peculiar topic described by *questionnaire*, *data privacy*, *work*, *bird flu*, and *wife*, but at least 12 of the topics could still be mapped onto the solution by visual inspection. This indicates that, given the chosen number of topics, priors that fell within a reasonable range did not substantially affect the conclusions.

### Correlational analyses

#### Word-level analyses

**Fig 9:**
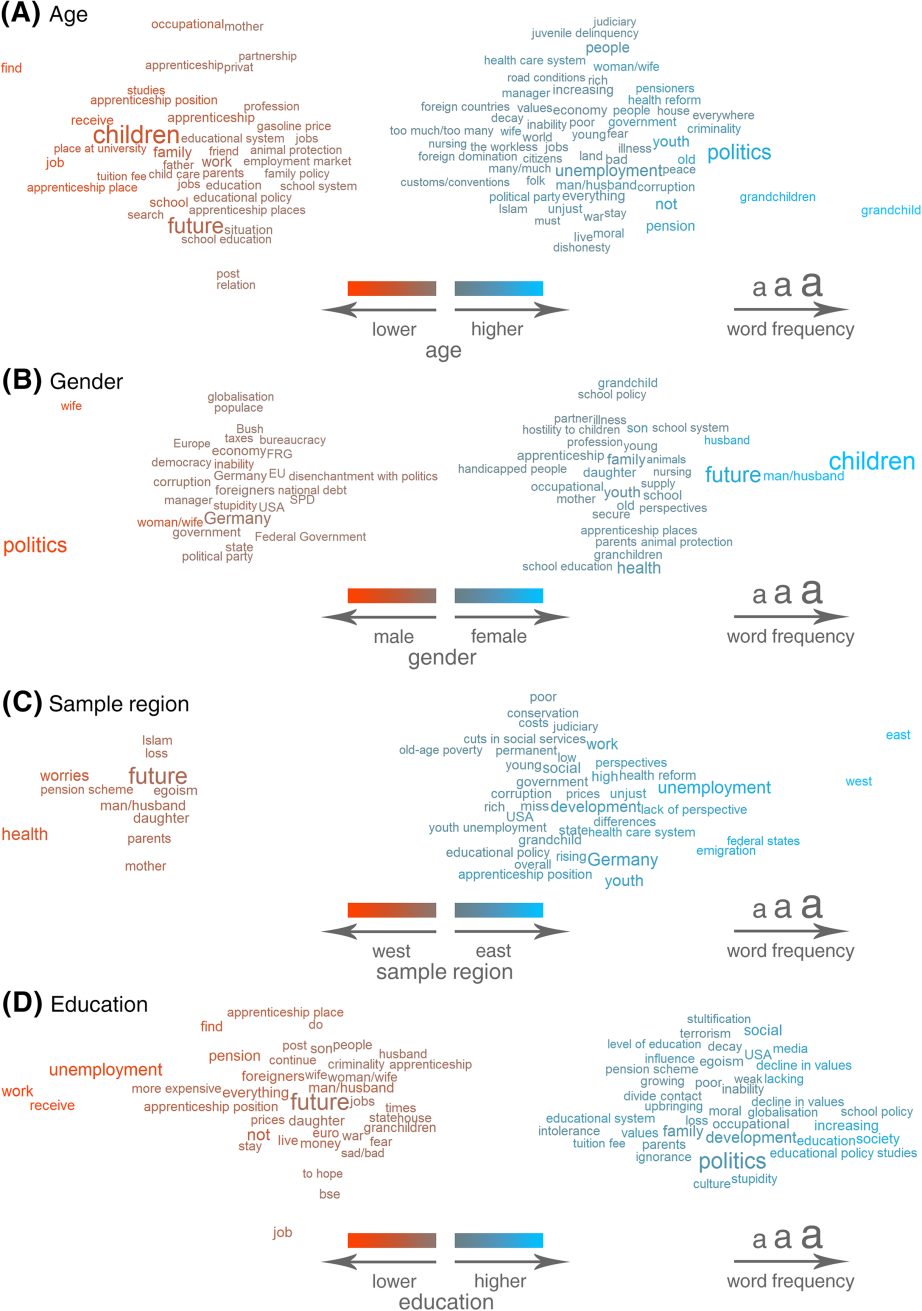
Results of word-level correlational analyses linking the use of single words with features of the respondents within the subsample who answered the open-ended question. Size reflects the frequency of the word across all answers; horizontal position and color reflect both the strength and direction of the associations; all displayed words are significant at *p* < .05 (Bonferroni-corrected). (A) Results of linear regressions predicting age. (B) Results of binary-logistic regressions predicting gender. (C) Results of binary-logistic regressions predicting sample region. (D) Results of ordered logistic regressions predicting education.

**Fig 10:**
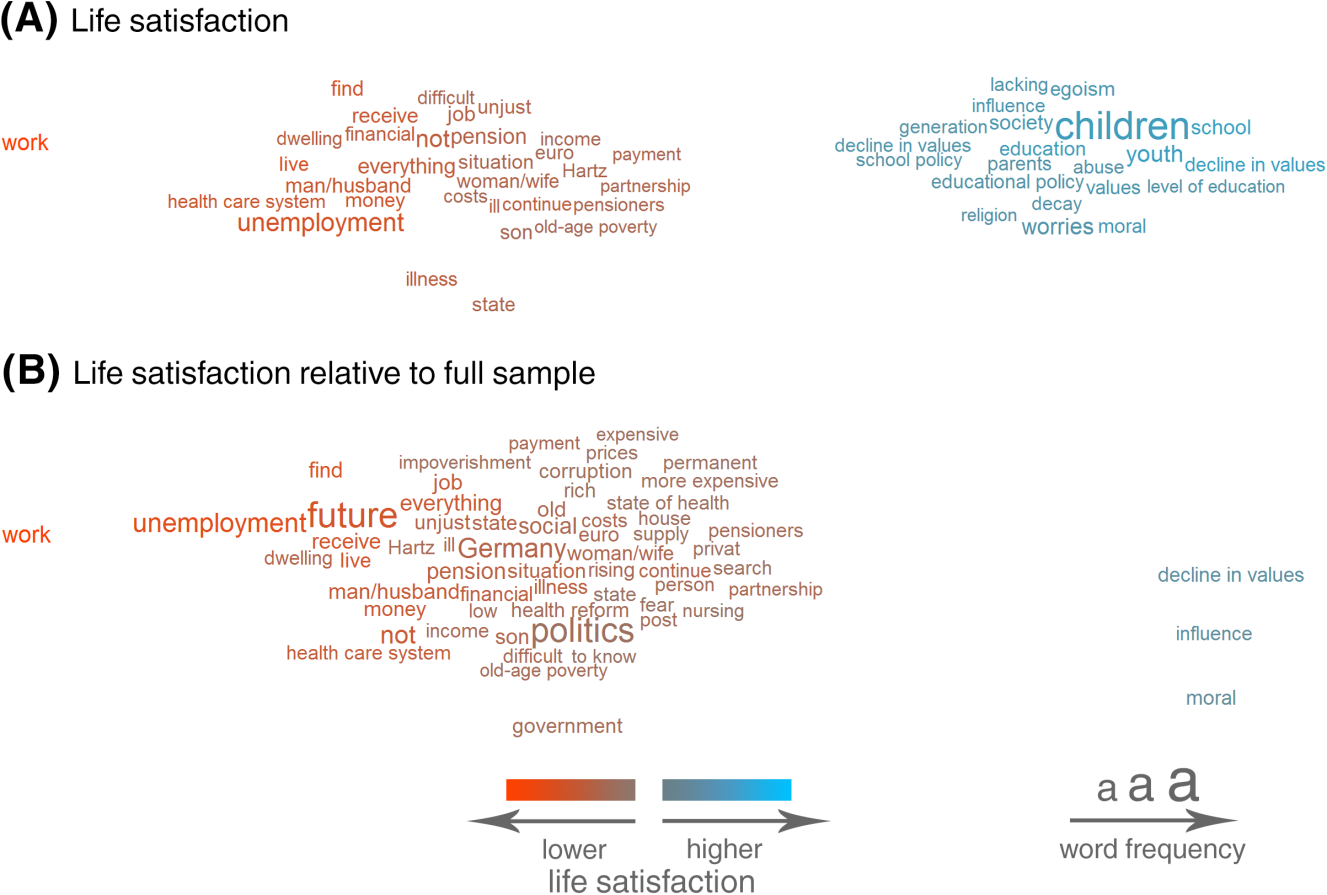
**Results of word-level correlational analyses linking the use of single words with life satisfaction (A) in the subsample that answered the open-ended question and (B) relative to the full sample.** Size reflects the frequency of the word across all answers; horizontal position and color reflect both the strength and direction of the associations; all displayed words are significant at *p* < .05 (Bonferroni-corrected).

How are the free-text answers linked to respondents’ features such as socio-demographic variables and personality? Results of correlational analyses between the use of single words and features of the respondent are displayed in word clouds in which size reflects the frequency of the word, and horizontal position and color both reflect the strength and direction of the association, see Figs 9 and 10. Words are displayed if the correlation between word use and the respective dependent variable met the statistical significance threshold of *p* < .05 (Bonferroni-corrected; accounting for the number of words, *N* = 243, tested per dependent variable). Word size increases with frequency in a monotonic but non-linear fashion to keep the figures readable despite the large range of word frequencies.

Consider, for example, the words that were correlated with age (within the subsample of respondents who provided a textual answer), Fig 9A. The words that showed the strongest positive association with age–*grandchild* (“Enkel”) and *grandchildren* (“Enkelkinder”), close to the right edge of the figure–seem highly plausible given that age increases the chances of having grandchildren. However, we can also see that a wide range of political and societal worries (e.g. regarding *politics*, *unemployment*, *government*, *youth* as in youth unemployment, *moral*, and *dishonesty*) were associated with older age. On the left side of the plot, we see, by contrast, words that were negatively correlated with age (i.e. that were more typical of younger respondents). The younger respondents in the sample seemed to be more concerned about their employment and future prospects (*find*, *job*, *apprenticeship place*, *tuition fee*) and also about their *children* and *family*.

Other interesting patterns emerged, for example, between sample region and worry words. Whereas only a handful of words mostly referring to the private sphere (e.g. *health*, *future*, *parents*) were indicative of West German respondents, East German respondents spoke or wrote more about structural issues such as *unemployment*, *emigration*, and *development*. *East* and *west* showed the highest association with the sample region East Germany, and a cursory string search revealed that these two words often occurred together in textual answers that addressed disparities between East and West Germany.

Thus far, we have presented the results of analyses that included only respondents who answered the open-ended question. However, in the presence of selection effects, the strength of correlations between words and other variables sometimes differed when individuals who did not answer the question were included. To demonstrate such discrepancies, Fig 10 shows the results of correlational analyses of life satisfaction (A) in the subsample that answered the question and (B) in the full sample, thus including respondents who filled out the survey but did not answer the open-ended question.

The analyses in the subsample that answered the question (Fig 10A) show that the same number of worry words were negatively and positively correlated with life satisfaction. Words that express worries about the circumstances of living and financial security were negatively associated with life satisfaction (e.g. *work*, *unemployment*, *job*, *financial*, *dwelling*). But the use of the word *children* or worries about values (*decline in values*, *moral*, *values*, *egoism*) seemed to be indicative of higher life satisfaction. The same analysis run on the full sample (i.e. including respondents who did not answer the open-ended question) offered quite a different picture (Fig 10B). Here, the words that were negatively correlated with life satisfaction vastly outnumbered the words that were positively correlated. This discrepancy can be attributed to the selection effects that led to differences between individuals who answered the open-ended question and individuals who did not answer the question. The individuals who used the word *children* might have been more satisfied with their lives in comparison with the people who answered the question but wrote about something else. However, they were not more satisfied with their lives than the average of the complete sample, including the large number of people who did not provide any answer to the question, who were more satisfied overall than the people who did answer. Such predictable discrepancies arose whenever the dependent variable of interest was significantly associated with response behavior (i.e. significant effects in the analysis of selection effects).

Correlational analyses between the use of single words and the Big Five personality traits yielded only a few significant results except for openness to experience, where we observed positive correlations with words regarding worries about politics and society (e.g. *politics*, *people*, *decline in values*, *intolerance*) and negative correlations with words regarding, for example, the employment situation (*unemployment*, *work*) and family members (*wife*, *husband*, *children*). A visual representation of the word-level correlational analyses of the personality traits can be found in S1 Fig.

Numerical results of all correlational analyses on the word level (including results of analyses on the full sample not pictured here) can be found in S1 Table.

#### Topic-level analyses

**Fig 11:**
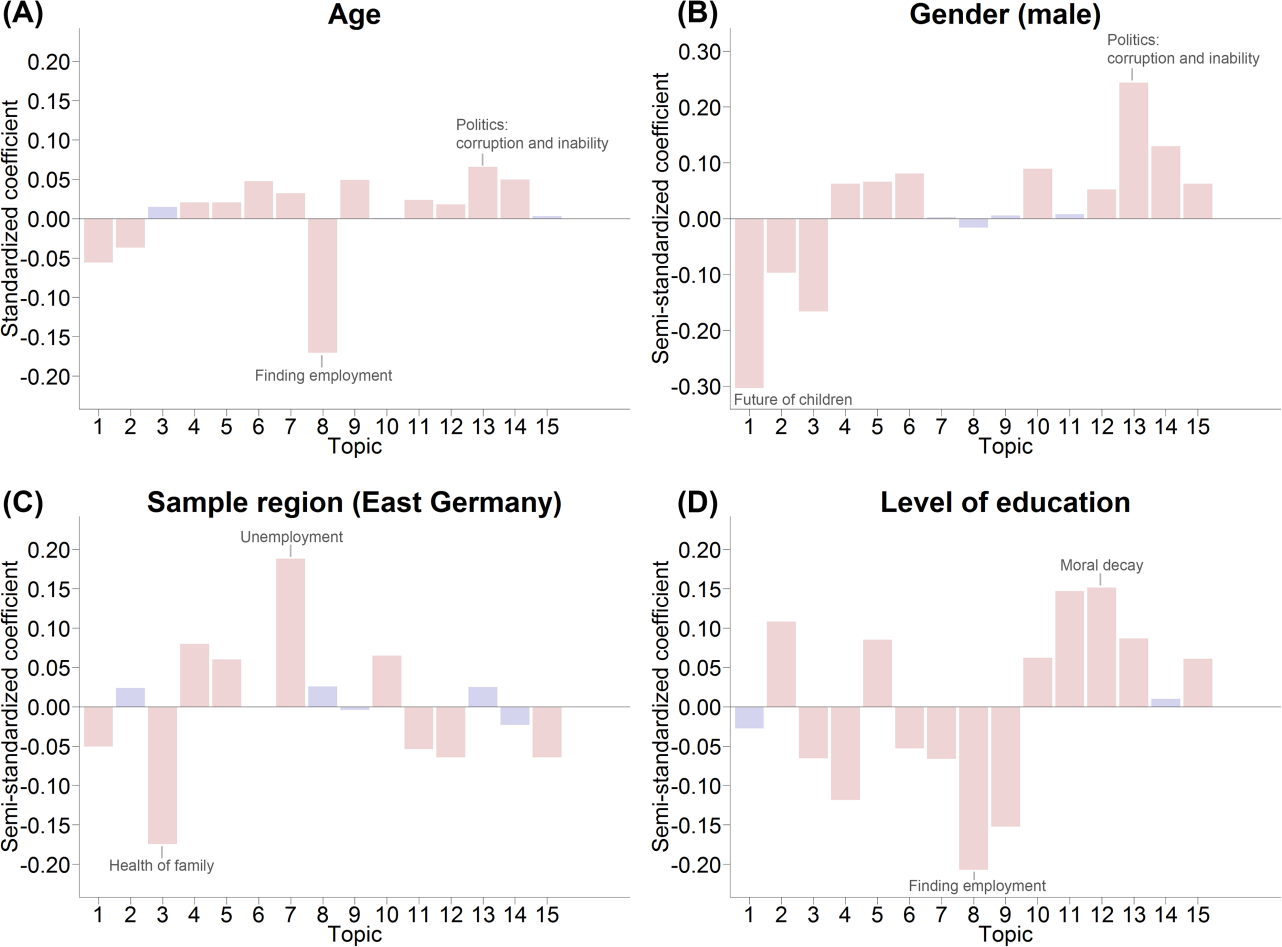
Results of correlational analyses linking features of the respondent and topic occurrence in the textual answer within the subsample that answered the open-ended question. Topics with the highest and lowest coefficients are labeled for each feature; red bars indicate significant results at *p* < .05 (Bonferroni-corrected). (A) Results of linear regressions predicting age. (B) Results of binary-logistic regressions predicting gender. (C) Results of binary-logistic regressions predicting sample region. (D) Results of ordered logistic regressions predicting education.

**Fig 12:**
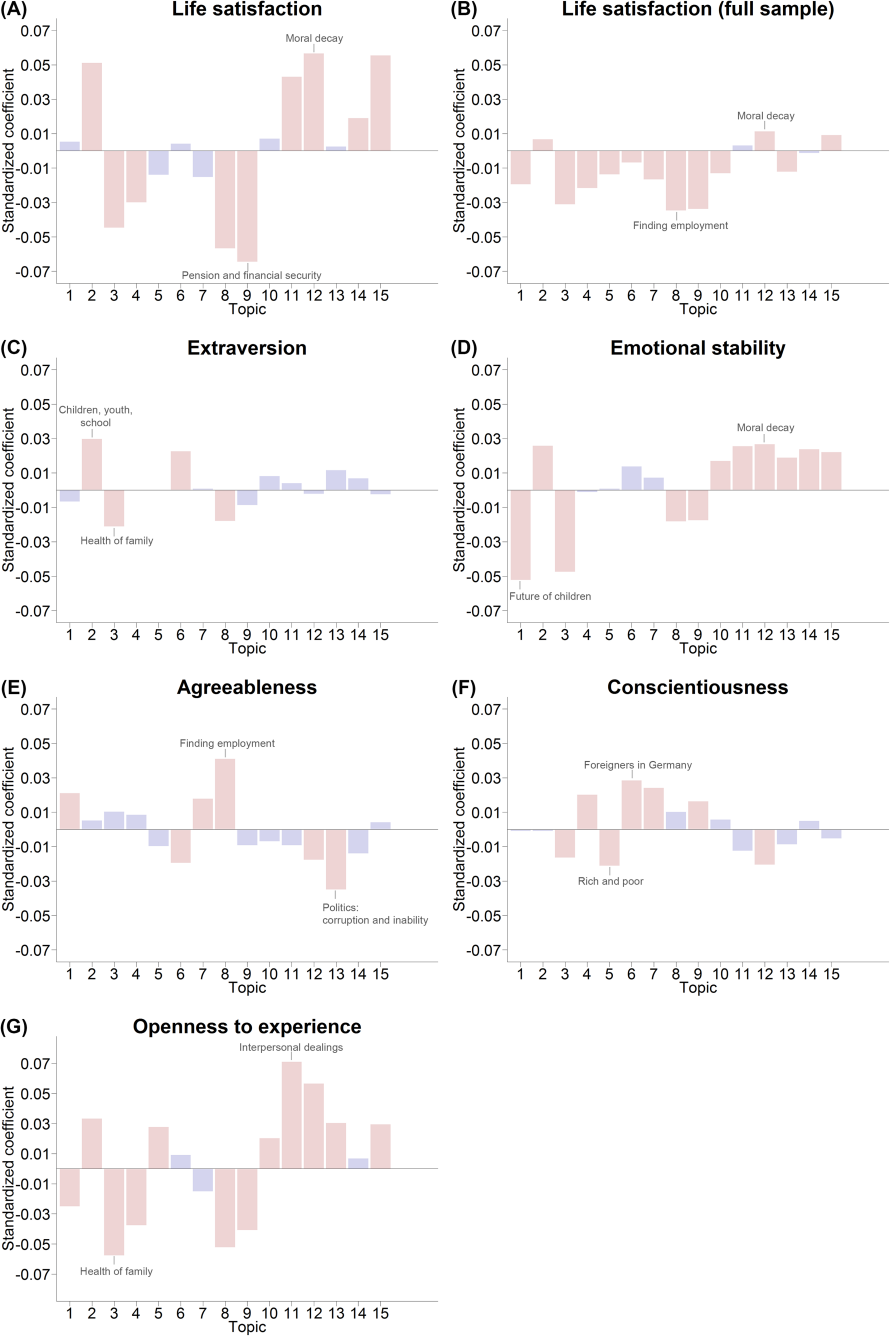
Results of correlational analyses linking the personality of the respondent and topic occurrence in the textual answer. Topics with the highest and lowest coefficients are labeled. Red bars indicate significant results at *p* < .05 (Bonferroni-corrected). (A) Results of the linear regressions predicting life satisfaction in the subsample that answered the open-ended question. (B) Results of the linear regressions predicting life satisfaction in the full sample, including respondents who did not answer the open-ended question. (C)-(G) Results of the linear regressions predicting the Big Five personality traits in the subsample that answered the open-ended question.

Figs 11 and 12 show the results (i.e. the regression coefficients) of the topic-level correlational analyses. As described earlier, these analyses included topic occurrence as the independent variable but otherwise used the same model as the word-level analyses. Coefficients are highlighted in color if they were significant at *p* < .05 (Bonferroni-corrected; accounting for the number of topics, *N* = 15, tested per dependent variable); the topics with the highest and lowest coefficients are labeled by dependent variable.

Regarding relationships between socio-demographic variables and worries reported in the textual answers on a topic level (Fig 11), in general, the results confirmed the patterns from the word-level analyses yet offered a higher degree of abstraction. For example, we again found that worries about employment (Topic 8) were more prevalent in the younger respondents (Fig 11A), whereas worries about politics (Topic 13, Topic 14) were associated with older ages. The analysis of sample region and worry topics (Fig 11C) showed that worries about unemployment (Topic 7) were more typical of respondents from East Germany, whereas the more private worries about the health of one’s family (Topic 3) were more typical of respondents from West Germany.

Not only did the results of analyses predicting life satisfaction (Fig 12A and 12B) show the same trends as the word-level correlational analyses, but they again demonstrated selection effects. In the subsample that answered the open-ended question, we again found that worries about employment (Topic 8) and worries about pension and financial security (Topic 9) were distinctly negatively related to life satisfaction, whereas topics about moral decay (Topic 12) showed positive associations with life satisfaction. However, when we extended the analyses to include respondents who did not answer the open-ended question, the positive associations with life satisfaction disappeared or became strongly attenuated. Certain results also went beyond the word-level analyses. We found that worries about war and terrorism (Topic 15) were positively correlated with life satisfaction (in the subsample as well as in the full sample) even though none of the most important words of this topic were visible in the word-level analysis of life satisfaction (Fig 10), thus demonstrating that different degrees of abstraction can lead to the discovery of different associations.

While word-level analyses of the Big Five personality traits led to rather sparse results (S1 Fig), topic-level correlations revealed a multitude of statistically significant associations (Fig 12C–12G). For example, we found that worries about the future of children (Topic 1) and the health of one’s family (Topic 3) were associated with lower levels of emotional stability (even after accounting for confounding with gender), whereas Topics 10 to 15 –all revolving around political or societal issues–were associated with higher levels of emotional stability.

## Discussion

### Selection effects

The analysis of selection effects revealed that whether or not participants responded to the open-ended question depended on the survey mode and multiple characteristics of the respondents. From a conceptual point of view, we can distinguish three sources that contribute to whether or not a respondent will answer an open-ended question: The question, the respondent, and the interplay between the respondent and the question.

Because we analyzed only one question, we could not determine whether the content of the question had an influence on whether or not people would answer it during the course of a social survey. However, we were able to analyze effects of the mode of the question and found that respondents were most likely to answer in the oral mode, indicating that people are less likely to skip an open-ended question in a face-to-face interview situation, whereas they might ignore a small field for entering text at the very bottom of a questionnaire when they fill out a survey by themselves.

The open-ended question was answered more frequently by individuals with a higher level of education, an effect that was also found in the Australian Longitudinal Study on Women’s Health [39] and in the Audit Commission Study of Recent Mothers [40]. This finding might thus reflect that more educated respondents are more likely to answer open-ended questions, regardless of the content of the specific question. Respondents with a lower level of education might not deem their answers important enough to be of interest to the researchers, or they might simply be less cooperative in the survey situation. Beyond the effect of education, higher scores on openness to experience increased response rates as well. Both effects are compatible with Garcia et al.’s speculation that respondents might be more or less articulate, resulting in a higher or lower preference for the open-ended format [40]. Furthermore, first-generation immigrants answered less often, probably because of language barriers; second-generation immigrants, however, did not differ from respondents without an immigration background in their response behavior. A small positive association was found between extraversion and free-text answers. People who describe themselves as talkative (a subdimension of extraversion) might also show a greater need to communicate in an interview situation or maybe even on a questionnaire.

Furthermore, there were a number of associations between the characteristics of the respondent and their answering behavior that we attribute to the specific question asked in this study. In our sample, individuals who were less satisfied with their lives or less emotionally stable most likely had more worries to report in the first place or a greater need to report their worries, leading to higher response rates. The negative association between emotional stability and reporting worries can also account for the gender differences in the probability of answering the open-ended question: Women, on average less emotionally stable, were more likely to respond to the open-ended question, but the effect disappeared in the model that also accounted for the Big Five personality traits.

An interesting finding is that within- and between-subjects effects of respondents’ age differed in their direction. One explanation could be that older individuals had a preference for reporting their worries in an open-ended question (because they generally preferred the open-ended format or because they had more worries to report), but the repetitive nature of the survey–being asked the same questions year after year–might have decreased their motivation to answer. One woman, for example, earnestly answered the open-ended question 6 years in a row. In the seventh year, however, she stated: “After all, nobody is interested in my everyday worries.”

Putting these findings together, open-ended questions are likely to be answered by a non-representative subsample, a circumstance that has been considered an inherent limitation of free-text comments in surveys [40]. However, this does not render the data useless. Qualitative research normally does not aim for large random samples; rather, the aim is to judge–on the basis of contextual background variables–whether the hypotheses in question can be applied in other contexts [41]. More important, large representative surveys and panels allow for the explicit analysis of selection effects, an advantage over, for example, the analysis of online posts and comments where the part of the population that does not provide texts remains invisible.

In the case of the data we analyzed, one should keep in mind that the occurrence of a certain topic such as politics does not necessarily reflect the importance of this domain in the general population but might be an overestimation because respondents with a higher level of education are more likely to answer the open-ended question in the first place. Furthermore, correlations between word use and individual features might differ for the unobserved part of the sample that did not answer the open-ended question. Hypotheses derived from the free-text comments should thus be carefully re-examined and additionally validated in different research designs.

### Topic detection

What else are respondents worried about? The topics that emerged included the future of children to rising prices, unemployment, the gap between rich and poor, and the state of society including a decline in values. Some of these topics had not been covered by a corresponding closed-ended question in our sample (e.g. “Future of children,” “Gap between rich and poor”), thus demonstrating one of the advantages of bottom-up data analysis: The results can provide hints about topics that are important to respondents but were not included in the questionnaire.

Other topics substantially overlapped with the worries already covered with closed-ended questions. For example, the topic “Rising prices” overlapped with worries about one’s own financial situation and worries about the economy in general, both already included in the questionnaire; the topic “Foreigners in Germany” overlapped with the item asking for worries about immigration to Germany. This overlap allowed us to investigate whether answers to the open-ended question converged with answers to the closed-ended questions, and this can be seen as a test of convergent validity. For example, the strongest relationship between worries in the two modalities can be found between the already mentioned topic “Foreigners in Germany” and the item “immigration to Germany,” and other pairs of topics and items showed equally plausible relationships.

However, even in the case of overlap, the derived topics provided further information beyond the closed-ended questions. Respondents might choose to answer an open-ended question to emphasize a point that is of greater importance to them (e.g. rising prices and not economic growth in general); they might also want to further elaborate on the answers they provided in the closed-ended questions and add a certain sentiment. For example, we found two topics that centered around politics: “Politics,” which included names of politicians and parties, and “Politics: corruption and inability.” Adding a single item that asked for worries about politics would not have captured the wealth of derogatory terms found in the latter, which respondents used to describe politics (e.g. corruption, inability, dishonesty–among the top 10 words defining the topic, but followed by many more) and which expressed a very strong sentiment toward politics.

It is interesting that one of the topics turned out to capture words that referred to catastrophes in the broadest sense, including war, George W. Bush, Kosovo, bird flu, BSE and Islamism, as well as nuclear power. This might be an artifact caused by the co-occurrence of certain world events within the same year, but it might also reflect a tendency of certain individuals to list multiple current events that share a certain feature such as a high level of perceived threat. The prevalence of this topic corresponded to large international conflicts; whereas the prevalence of the topic “Rising prices” peaked after the financial crisis. These plausible results suggest that the automatically derived topics indeed capture worry complexes that are associated with actual world events.

All in all, topic modeling led to coherent topics that highlighted worry domains that had not been considered in the choice of items and provided additional insight into what respondents considered worth mentioning. Topic occurrence was linked to both worries reported in closed-ended questions and major world events, which hints at the validity of the derived topics.

#### Alternative approaches for detecting the underlying structure of textual data

Regarding the topic model used in this study, several alternative approaches would have been justifiable. For example, in an attempt to sort respondents’ answers into disjunct partitions, we could have chosen a clustering approach instead. We considered the topic model more sensitive to the nature of the answer (i.e. many respondents reporting more than just one issue in their answers), but this decision certainly depends on the specific situation in question.

Furthermore, we could have considered different topic modeling algorithms instead of LDA. Latent Semantic Analysis (LSA; based on either single vector decomposition, SVD, [42] or non-negative matrix factorization, NMF [43]) can be employed to identify latent concepts for document categorization on the basis of the decomposition of the term-document matrix. In general, the different models deliver similar results, but one may be more defensible in certain situations. A comparison of LDA and LSA by SVD or NMF [44] found that LDA generally produces more coherent topics than other algorithms and therefore “warrants strong consideration in applications in which human end-users interact with learned topics,” whereas SVD is preferable for document classification.

#### Potential for further validation

In studies in which the main objective is to detect the underlying structure of the documents–either by topic modeling or by clustering–more systematic approaches to validation are desirable to ensure that the results are useful for future research. Such a validation should be problem specific, and it will often rely on human ratings [45].

In one paradigm that has been used to evaluate the quality of topic models [34], human raters were presented a list of words from one topic and had to detect a randomly added unrelated word (word intrusion). In a second task, they were presented a document and a list of topics and had to detect a randomly added unrelated topic (topic intrusion). The participants’ performance in such tasks can be used to estimate how well the derived topic model matches human understanding.

Grimmer and King [13] used a different paradigm that also relies on human ratings to evaluate the results of their clustering algorithm. Raters were presented documents drawn from the same or from different clusters and had to rate the similarity of the documents. These similarity measures were then used to derive a numerical measure of cluster quality. Furthermore, they attempted to evaluate the usefulness of different clustering methods with respect to their potential for new discoveries. For this task, they relied on experts who performed pairwise comparisons of different clusterings to evaluate their informativeness.

### Correlational analyses

Linking features of the respondents to words and topics in the textual answer led to a wealth of results. In the following, we will discuss some of the central findings.

#### Socio-demographic variables and worries

Worry words and topics were related to socio-demographic variables such as age, gender, and education. This is highly plausible because, after all, worries capture what is relevant in respondents’ lives: Whereas younger people worry about how to *find* a *job* and *children*, older people are worried about their *pension* and *grandchildren*. On a topic level, younger people are concerned about “Future of children,” “Children, youth, school,” as well as “Finding employment,” whereas “Pension and financial security” are in the domain of older respondents. The results also offer insight into differences between the two sample regions of East and West Germany: While the cloud of words characteristic of West German respondents included worries that were more closely related to private life (e.g. *children*, *health*, *parents*, *husband*, and *wife*), East Germans wrote more about political and social issues (e.g. *unemployment*, *emigration*, *youth*, *health care system*). In the closed-ended item *Worries about immigration to Germany*, respondents from East Germany reported slightly but significantly more worries (Cohen’s *d* = 0.13, *p* < .001). By contrast, in the open-ended question, worries about *Islam* and *foreign domination* were more characteristic of West German respondents, and the topic “Foreigners in Germany” indicated no differences between the two parts of Germany. Thus, while East German respondents were more likely to *confirm* worries about immigration when asked about them, they were *not* more likely to *write* about immigration, foreigners, or Islam when asked about “any other worries.” One possible interpretation is that the topic was simply less salient in East Germany during the survey years we analyzed. For example, in 2011, foreigners made up less than 3% of the population of the Federal states in East Germany, while the Federal states in West Germany ranged from 5 to more than 10 percent according to Destatis, the Federal Statistical Office of Germany [46].

#### Life satisfaction and worries

People reporting worries about *work* or *unemployment* were less satisfied with their lives on average. This finding is in line with previous findings that unemployment has a lasting impact on life satisfaction [47] but also with the notion that the *fear* of losing one’s job is burdensome. At the other extreme, certain worries were positively related to life satisfaction, such as worries about the topic “moral decay.” One (admittedly trenchant) interpretation could be that people worrying about abstract concepts such as the decline of society probably lack more menacing issues to worry about; however, other confounding variables might also explain these findings. Positive correlations with life satisfaction in the full sample were less pronounced or disappeared completely because the full sample was on average more satisfied than the subsample that answered the open-ended question.

#### Big Five personality traits

Correlations between personality traits and worries were less pronounced overall than correlations with socio-demographic variables or life satisfaction. We observed only a few significant correlations in the word-level analyses; however, more effects yielded significance in the topic-level analyses. For example, worries about politics and societal issues such as moral decay were consistently associated with higher levels of emotional stability, whereas lower levels of emotional stability were instead associated with worries about one’s family. One possible interpretation is that low emotional stability is associated not only with more worries but also with worries that are “closer to home” and less abstract than worries about the current state of society or politics in general. However, other correlations are less easily interpreted, such as the relationship between higher levels of agreeableness and worries about employment and between higher levels of conscientiousness and greater worries about foreigners in Germany; and we did not test whether third variables (including social desirability as personality was measured via self-report) could account for any of these correlations.

#### Word-level analyses versus topic-level analyses

Which type of analysis is more suited to investigate links between free texts and other variables–a word-level analysis or a topic-level analysis? Word-level correlations led to a large number of strongly compartmentalized analyses, which raises the issue of alpha inflation but also makes the results harder to overlook and integrate. However, linking manifest answering behavior and other variables of interest is straightforward in word-level analyses: The coefficients simply reflect the difference in the dependent variable between people who used that word and people who did not use it. This connection is not so obvious for topic-level analyses. In the case of topic-level analyses, the coefficients do not directly reflect manifest answering behavior but the underlying topic assumed in the textual answer. A valid interpretation of a correlation between a topic and a variable of interest thus depends on the properties of the modeled topics (e.g., their validity and their homogeneity). However, topic correlations also offer a higher level of abstraction, which might be desirable for many research questions. Furthermore, our results regarding worries and the Big Five personality traits suggest that topic-level analyses might achieve greater statistical power because they bundle multiple related terms.

We thus recommend that both types of analyses be applied because they allow the data to be explored from different perspectives. If word-level results and topic-level results converge, the trust in the underlying pattern increases. If the analyses do not mutually support each other or even lead to contradictory conclusions, researchers should carefully consider various explanations for this finding. For example, word-level analyses can lead to “spurious” correlations that are driven by idiolects, whereas topic-level correlations become hard to interpret when a derived topic lumps together multiple issues.

### General discussion

#### Can we generalize results of automated text analysis?

An important limitation of the analysis of free-text comments is that we do not yet know whether and to what extent the results allow conclusions that apply to the whole sample to be drawn. In our case, the subsample that answered the open-ended question was not representative of the sample that took the survey; it differed on variables such as education, which might lead to a gross misrepresentation of worry topics. In the worst case scenario, there are certain worries that are highly specific to a part of the population that simply does not answer open-ended questions at all, and we would therefore miss that topic no matter how large our sample was originally. But even assuming that representativity was granted, it remains impossible to estimate the prevalence of a certain worry. The occurrence or absence of a topic is not as easily interpreted as the ticking of a specific box as an answer to a closed-ended question. This has been described as the “present-absent problem” [4], and it is also relevant in, for example, analyses of interview transcripts. The presence or absence of a word or a topic in a textual answer can have multiple meanings, and while an automated text analysis might rule out some of them (e.g. it is unlikely that an automated analysis will miss a keyword given that it was used in the text), it also introduces new aspects (e.g. a word might be considered absent because respondents chose a synonym that is not frequent enough to be visible in the analysis) and cannot decide whether, for example, a topic was forgotten or was actually not very relevant to the respondent or whether a topic was actually important to the respondent or simply very salient at that moment.

While differences in topic salience can be informative and can yield new research questions, they can also be introduced by the context of the survey. For example, a quick keyword search revealed at least 30 instances of people worrying about the survey itself, including worries about the questionnaire, its length, what will become of the responses, and even worries about “whoever came up with these questions.” One person even worried that she was not able to provide exact answers to the interviewer. These method artifacts might tell us something about the survey process, but they certainly do not provide unbiased insights into what people worry about on the days on which they are not surveyed. Furthermore, space limitations restricted respondents from reporting multiple answers, even if they had wanted to address multiple issues. However, one might also view this as an advantage over closed-ended questions because open-ended questions with space limitations force respondents to focus on what they consider most important.

#### The impact of data pre-processing and the objectivity of automated text analysis

Another important issue is that data preprocessing affects the data input and the analyses and could thus impact the results and the corresponding conclusions. For example, we decided to stem words that inevitably led to a loss of information on the used parts of speech; this step is, however, appropriate and necessary when one is not interested in linguistic features and focuses on semantics (i.e. content) in a highly inflected language such as German. On the other hand, we decided not to split compound words because, in the context of worries, the compound carries a meaning that goes beyond the isolated lexemes. Worries about “Jugendarbeitslosigkeit” (*youth unemployment*) were thus not split into worries about *youth* and worries about *unemployment*; worries about “Gesundheitswesen” (*health care system*) were not decomposed into worries about *health* (“Gesundheit”) and worries about “Wesen” (meaning *business* or *system* when used in a compound word). This approach, however, also led to some undesirable results, e.g. “Ausbildungsplatz” (literally *apprenticeship* + *place*) and “Ausbildungsstelle” (literally *apprenticeship* + *position*) turning up as distinct words, even though they could be considered synonyms. One approach would be to adjust the decomposition of compounds manually by setting up lists of words that should or should not be split given the research question at hand. However, one could argue that such a procedure in turn decreases objectivity and can become highly effortful and thus partly undermines the idea of an automated analysis. When analyzing English textual data, the problem takes on another form: Compounds that one might want to analyze as units such as *youth unemployment* get split in the process of tokenization; in this case, one would prefer not only to analyze single words (so-called unigrams) but also to analyze sequences of, for example, two to three words (bigrams and trigrams).Analyzing textual data requires researchers to make multiple decisions about how to pre-process and analyze textual data. Some might depend on the response language and features of the texts to be analyzed; some might depend on the research question of interest, that is, the aim of the analyses; and last but not least, some might depend on the presuppositions and judgments of the researcher. Even the decision *not* to pre-process the data and simply to assemble a list of words to be counted carries the implicit presupposition that the words on the list are the words of interest for the research question. An automated text analysis comes with a large number of so-called researcher degrees of freedom [48]. Accordingly, it is susceptible to well-known scientific nuisances: Researchers can cherry-pick the most appealing results or adjust the pre-processing and parameters of the analyses until the results fit the desired outcome. Thus, we consider it crucial that researchers disclose all the steps used in the data pre-processing and the analyses and provide the rationale behind them. Greater transparency also allows researchers to understand that statistical methods developed by, for example, computer scientists are not black boxes that are fed data without any additional assumptions and magically return objective knowledge. Rather, they are algorithms that often require adjustment to yield the most insightful results given the data. Full transparency about the research process allows other researchers to understand and, if necessary, criticize the decisions made in the process of data analysis.

#### Depth of automated text analysis

The automated textual analyses we ran remained shallow in some respects, especially in comparison with intellectual analyses of qualitative interviews. This lies in the very nature of the data provided in this study, which were typical for open-ended questions included in larger social surveys (very brief statements, often only single phrases or words) but also in the analytical approach (e.g. a bag-of-words approach in the current study). There can be a profound loss of information through the use of a context-insensitive analyses in comparison with an intellectual analysis. For example, an answer such as “my wife does not want me to write about my soccer club” (which was actually in our textual data) provides information that cannot be reflected by a simple count of the words “wife,” “want,” “write,” “soccer,” and “club.”

In a review of natural language processing research, Cambria and White compare understanding a text through word-level processing to “attempting to understand a picture by analyzing it at pixel-level” and claim that even sophisticated frameworks, such as IBM’s question-answering system Watson, have no “understanding” of what they are doing [49]. The two authors envision the evolution from natural language processing to natural language *understanding* as a shift from the currently predominant word-based techniques to concept-based techniques and finally narrative-based techniques.

Current approaches to lexical semantics by means of word vector representations [50; 51] show promising results in this direction. Word vectors produced by word2vec [50] can be used to compute the semantic similarity between words. For example, it has been shown that in the resulting vector space, certain word relationships can be identified, such as *queen ≈ king–man + woman* [52]. One drawback of these models is the generally large amount of training data needed to achieve meaningful results. The usual training corpora include millions of sentences and billions of words. Thus, typical social science data sets are not large enough to train state of the art word2vec domain models on them. However, models trained on existing corpora (e.g. news corpora, Wikipedia text) may be used to introduce an external source of world knowledge. Word2vec models excel in the domain of word similarities and analogies. They are thus particularly suited for research questions that start from particular seed words of interest whose relationships to other words will be investigated.

While automated text analysis might eventually reach a level of natural language understanding, the technology is currently far from being suited to replace qualitative (i.e. intellectual, human-based) analyses of texts in a large number of applications.

#### Potential

Despite the technical obstacles to automated text analysis, and despite the methodological issues and current limitations, an automated analysis of free texts offers an attractive addition to quantitative research.

First, textual data are readily available in huge quantities–for example, from online sources such as Facebook, Twitter, and blogs, and, as in this study, from open-ended questions that have already been included in large-scale surveys and other studies but that have often not been analyzed to date. Data from social media have their own advantages (e.g. the sheer number of users, the interactive character of text messages, and the informal context) and have become increasingly popular in research; see [53] for a recent summary on how to integrate these data into psychological research. In comparison, data from representative social surveys and panel studies come with a large number of variables describing, for example, socio-economic circumstances in great detail and can be used to analyze who provided the textual data–and who did not. In both cases, the sheer sample size will often lead to a high level of statistical power. Although the analyses we presented in this paper were exploratory in nature, automated text analysis could also be used for more focused analyses and to test specific hypotheses in confirmatory investigations [54].

Second, the automated analysis of textual data opens new sources of information. According to Mehl and Gill [10], free texts share zero method variance with the commonly used self-report rating scales. The demands that open-ended questions put on respondents go beyond simply marking the correct answer, and thus, they prompt different cognitive processes and a different type of response behavior. Respondents are no longer confronted with a set of given answers and thus do not depend on what the designer of the questionnaire considers to be an exhaustive list of answer options. The open character of the question might motivate respondents to reveal more about what they consider important regarding a certain issue; results drawn from analyses of open-ended questions might thus lead to insights that go beyond the findings that come from closed-ended questions.

## Supporting information

S1 List List of common abbreviations used in open-ended text answers. (CSV)

S2 List Re-expansion word list. (CSV)

S3 List German to English translation of words displayed in figures. (CSV)

S1 Fig Visual representation of word-level correlational analyses of the Big Five personality traits. (TIF)

S1 Table Numerical results of the correlational analyses. (PDF)
