# Supplementary figures and images for: "What else are you worried about?" – Integrating textual responses into quantitative social science research
Source: PLoS One. 2017 Jul 31;12(7):e0182156. doi: 10.1371/journal.pone.0182156 (PMC5536367; doi:10.1371/journal.pone.0182156)

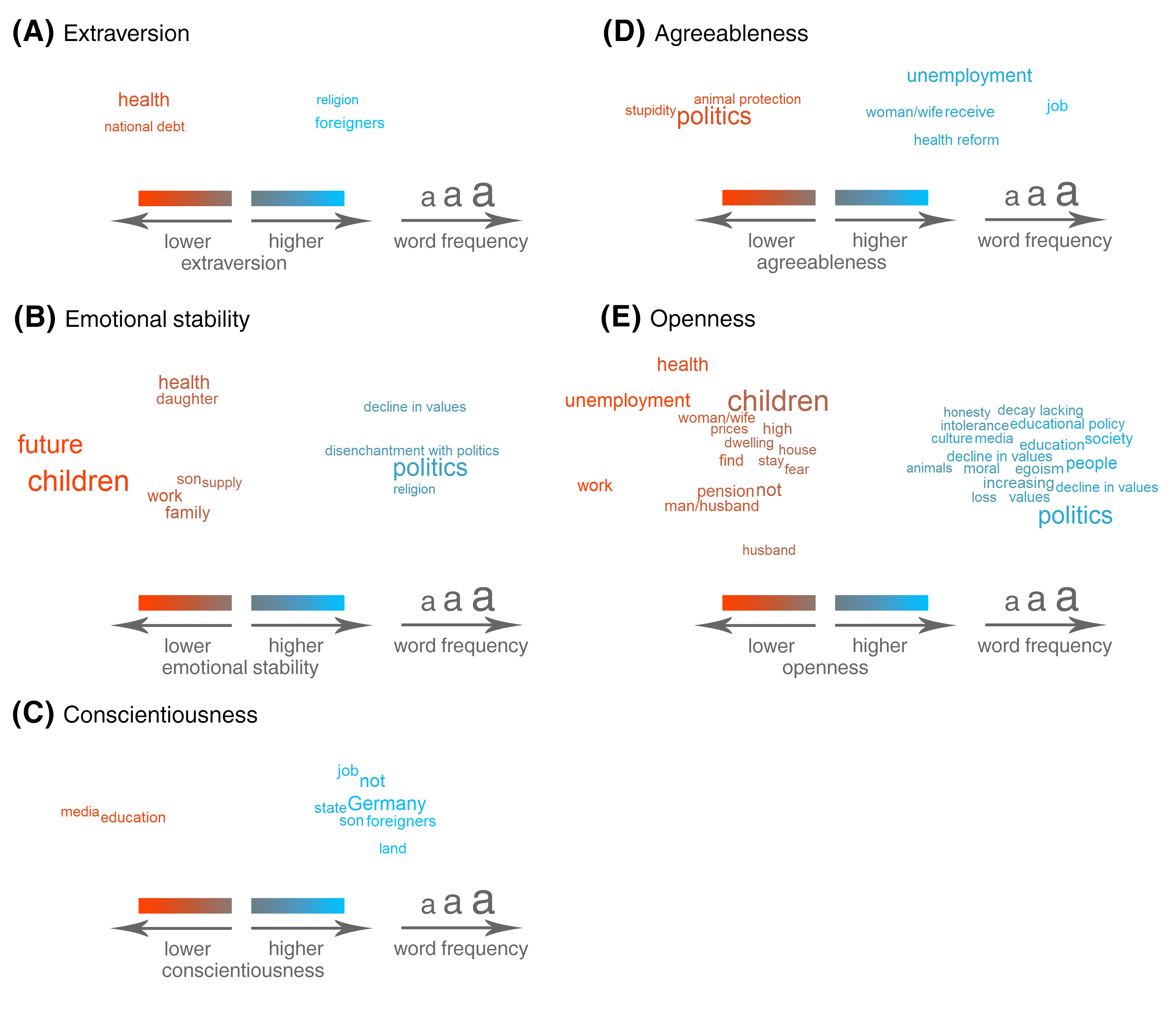

Supplement: S1 Fig — (TIF) [file pone.0182156.s004.tif]
