# Supplementary material for: "What else are you worried about?" – Integrating textual responses into quantitative social science research
Source: PLoS One. 2017 Jul 31;12(7):e0182156. doi: 10.1371/journal.pone.0182156 (PMC5536367; doi:10.1371/journal.pone.0182156)
Supplement: S1 Table — (PDF) [file pone.0182156.s005.pdf]

S1 Table. Numerical results of correlational analyses.

Results of correlational analyses of the subsample providing free text answers, dependent variable: sex (0 = male, 1 = female). Only words with $p < .001$ are displayed, numbers indicate semi-standardized regression regression coefficients

kinder	0.313
zukunft	0.217
politik	-0.281
arbeitslosigkeit	0.038
deutschland	-0.070
gesundheit	0.111
jugend	0.079
menschen	0.038
familie	0.087
gesellschaft	-0.038
sohn	0.108
wirtschaft	-0.066
ausbildung	0.062
alt	0.095
schule	0.083
zunehmend	-0.036
tochter	0.100
mann	0.284
ausländer	-0.048
sicher	0.046
beruflich	0.074
deutsch	-0.062
regierung	-0.062
korruption	-0.067
staat	-0.065
enkel	0.091
jung	0.052
unfähigkeit	-0.113
frau	-0.190
gesundheitsreform	0.039
eltern	0.063
enkelkinder	0.079
krankheit	0.049
partei	-0.053
mutter	0.050

usa	-0.054
gewalt	0.040
globalisierung	-0.060
perspektiven	0.060
beruf	0.047
angst	0.040
bevölkerung	-0.044
dummheit	-0.096
steuern	-0.066
brd	-0.042
schulbildung	0.062
eu	-0.061
versorgung	0.067
ausbildungsplätze	0.073
kinderfeindlichkeit	0.044
demokratie	-0.059
verlogenheit	-0.038
manager	-0.055
infaltion	-0.041
ehemann	0.211
reform	-0.041
bush	-0.051
bürokratie	-0.053
islam	-0.042
tierschutz	0.083
schulsystem	0.055
ehefrau	-0.257
behinderte	0.047
benzinpreise	-0.042
wohlergehen	0.040
bundesregierung	-0.063
staatsverschuldung	-0.064
schulpolitik	0.049
politikverdrossenheit	-0.067
partner	0.050
betreu	0.044
überfremdung	-0.040
spd	-0.054
europa	-0.046
tiere	0.089
arroganz	-0.040
pflege	0.052

Results of correlational analyses of the complete sample, dependent variable: sex (0 = male, 1 = female). Only words with $p < .001$ are displayed, numbers indicate semi-standardized regression regression coefficients

kinder	0.133
zukunft	0.091
politik	-0.087
arbeitslosigkeit	0.018
deutschland	-0.019
gesundheit	0.046
jugend	0.032
sorgen	0.014
menschen	0.017
familie	0.038
sohn	0.044
wirtschaft	-0.021
ausbildung	0.028
alt	0.036
schule	0.036
tochter	0.040
mann	0.117
ausländer	-0.015
situation	0.014
sicher	0.020
beruflich	0.032
deutsch	-0.020
regierung	-0.022
korruption	-0.023
staat	-0.021
enkel	0.033
jung	0.021
unfähigkeit	-0.039
frau	-0.066
gesundheitsreform	0.015
eltern	0.027
enkelkinder	0.028
bildungspolitik	0.015
krankheit	0.019
partei	-0.019
mutter	0.021
usa	-0.018
gewalt	0.017

globalisierung	-0.020
perspektiven	0.024
beruf	0.020
angst	0.016
bevölkerung	-0.015
armut	0.014
ausbilungsplatz	0.015
dummheit	-0.033
steuern	-0.022
brd	-0.014
schulbildung	0.025
eu	-0.021
versorgung	0.026
ausbildungsplätze	0.030
kinderfeindlichkeit	0.018
demokratie	-0.020
manager	-0.020
infaltion	-0.014
ehemann	0.085
reform	-0.014
bush	-0.017
bürokratie	-0.018
islam	-0.015
tierschutz	0.034
schulsystem	0.023
ehefrau	-0.088
behinderte	0.019
wohlergehen	0.016
bundesregierung	-0.022
staatsverschuldung	-0.022
schulpolitik	0.020
politikverdrossenheit	-0.024
partner	0.020
betreu	0.017
überfremdung	-0.015
kinderbetreuung	0.016
spd	-0.019
europa	-0.016
tiere	0.035
pflege	0.020

Results of correlational analyses of the subsample providing free text answers, dependent variable: age. Only words with $p < .001$ are displayed, numbers indicate standardized regression regression coefficients

kinder	-0.067
zukunft	-0.047
politik	0.080
arbeitslosigkeit	0.039
nicht	0.061
jugend	0.062
sorgen	0.020
menschen	0.038
arbeit	-0.037
familie	-0.055
rente	0.062
alles	0.031
bekommen	-0.078
hohe	0.019
finden	-0.096
arbeitsplatz	-0.087
wirtschaft	0.020
ausbildung	-0.050
alt	0.067
schule	-0.057
zunehmend	0.023
mann	0.040
ausländer	0.019
schlecht	0.023
situation	-0.020
beruflich	-0.050
leben	0.023
regierung	0.048
korruption	0.032
reich	0.021
enkel	0.111
bildung	-0.027
ungerecht	0.033
leute	0.037
arm	0.022
jung	0.027
krieg	0.022
unfähigkeit	0.021

frau	0.047
gesundheitsreform	0.046
eltern	-0.040
enkelkinder	0.089
welt	0.021
land	0.021
arbeitsplätze	0.022
moral	0.031
bildungspolitik	-0.030
krankheit	0.022
lehrstelle	-0.061
partei	0.037
werte	0.025
kriminalität	0.043
mutter	-0.023
gesundheitswesen	0.034
bleiben	0.020
verfall	0.022
beruf	-0.033
angst	0.021
kosten	0.018
jugendarbeitslosigkeit	0.019
bürger	0.022
viel	0.025
studium	-0.069
ausbilungsplatz	-0.081
schulbildung	-0.024
gesamt	0.018
generation	0.019
ausbildungsplätze	-0.024
volk	0.025
krank	0.020
kinderfeindlichkeit	-0.018
bildungssystem	-0.028
haus	0.026
arbeitsmarkt	-0.020
gesundheitszustand	0.018
gesundheitspolitik	0.018
manager	0.032
frieden	0.029
privat	-0.024
arbeitslose	0.020
ehemann	0.018
zunahme	0.019

jugendkriminalität	0.024
islam	0.022
freund	-0.040
tierschutz	-0.024
rentner	0.051
beziehung	-0.026
schulsystem	-0.022
ehefrau	0.033
lehre	-0.037
ausland	0.020
benzinpreise	-0.039
job	-0.039
vater	-0.034
zuviel	0.020
arbeitsstelle	-0.031
unehrlichkeit	0.022
überfremdung	0.024
kinderbetreuung	-0.032
stellen	-0.025
suchen	-0.040
familienpolitik	-0.021
überall	0.021
europa	0.018
altersarmut	0.018
ehrlich	0.018
studiengebühren	-0.049
studienplatz	-0.061
justiz	0.023
brutalität	0.018
pflege	0.026
straßen	0.026
sitten	0.020
partnerschaft	-0.025
musst	0.020

Results of correlational analyses of the complete sample, dependent variable: age. Only words with $p < .001$ are displayed, numbers indicate standardized regression coefficients

kinder	-0.012
politik	0.040
arbeitslosigkeit	0.021

gesundheit	0.010
nicht	0.028
jugend	0.028
sorgen	0.013
menschen	0.019
arbeit	-0.007
familie	-0.015
sozial	0.007
rente	0.028
alles	0.016
allgemein	0.009
bekommen	-0.023
sohn	0.010
hohe	0.011
finden	-0.030
arbeitsplatz	-0.027
wirtschaft	0.012
ausbildung	-0.014
alt	0.027
schule	-0.017
zunehmend	0.013
tochter	0.008
mann	0.017
ausländer	0.011
schlecht	0.012
beruflich	-0.015
deutsch	0.007
leben	0.012
geld	0.009
regierung	0.021
korruption	0.015
staat	0.008
reich	0.011
egoismus	0.008
enkel	0.042
bildung	-0.006
ungerecht	0.015
leute	0.016
steigend	0.007
arm	0.011
jung	0.013
euro	0.007
krieg	0.011
unfähigkeit	0.011

frau	0.021
über	0.007
gesundheitsreform	0.019
eltern	-0.012
enkelkinder	0.034
welt	0.010
land	0.011
arbeitsplätze	0.011
moral	0.014
bildungspolitik	-0.008
krankheit	0.010
lehrstelle	-0.019
partei	0.016
ost	0.007
werte	0.012
kriminalität	0.018
gleichgültigkeit	0.008
lage	0.008
uns	0.008
gesundheitswesen	0.014
bleiben	0.010
verfall	0.011
beruf	-0.010
angst	0.010
kosten	0.009
jugendarbeitslosigkeit	0.009
bürger	0.010
bevölkerung	0.009
viel	0.011
studium	-0.023
ausbilungsplatz	-0.027
schulbildung	-0.007
gesamt	0.009
generation	0.009
ausbildungsplätze	-0.007
volk	0.011
krank	0.009
bildungssystem	-0.008
haus	0.011
gesundheitszustand	0.008
gesundheitspolitik	0.008
verlogenheit	0.007
manager	0.014
frieden	0.012

privat	-0.007
arbeitslose	0.009
perspektivlosigkeit	0.007
ehemann	0.008
zunahme	0.009
werteverlust	0.007
jugendkriminalität	0.010
islam	0.010
freund	-0.012
tierschutz	-0.007
rentner	0.020
beziehung	-0.007
schulsystem	-0.007
ehefrau	0.015
lehre	-0.012
denken	0.007
ausland	0.009
benzinpreise	-0.012
job	-0.012
vater	-0.011
untereinander	0.007
zuviel	0.009
arbeitsstelle	-0.009
unehrlichkeit	0.009
überfremdung	0.011
kinderbetreuung	-0.010
stellen	-0.007
suchen	-0.013
überall	0.009
europa	0.008
altersarmut	0.008
zuwanderung	0.008
ehrlich	0.008
studiengebühren	-0.016
studienplatz	-0.021
justiz	0.010
brutalität	0.008
nach	0.007
pflege	0.010
gross	0.007
straßen	0.011
sitten	0.009
partnerschaft	-0.008
musst	0.009

Results of correlational analyses of the subsample providing free text answers, dependent variable: sample region (0 = East Germany, 1 = West Germany). Only words with $p < .001$ are displayed, numbers indicate semi-standardized regression coefficients

kinder	0.040
zukunft	0.064
arbeitslosigkeit	-0.136
deutschland	-0.099
gesundheit	0.129
jugend	-0.100
entwicklung	-0.085
sorgen	0.113
arbeit	-0.089
sozial	-0.064
gesellschaft	0.046
hohe	-0.090
tochter	0.061
mann	0.074
regierung	-0.056
korruption	-0.055
fehl	-0.051
staat	-0.072
reich	-0.043
egoismus	0.049
enkel	-0.066
ungerecht	-0.092
steigend	-0.085
arm	-0.048
jung	-0.048
frau	0.050
über	-0.039
gesundheitsreform	-0.071
eltern	0.070
bildungspolitik	-0.050
lehrstelle	-0.087
ost	-0.189
mutter	0.072
usa	-0.063
gesundheitswesen	-0.098

erhalt	-0.047
preise	-0.055
perspektiven	-0.087
kosten	-0.058
jugendarbeitslosigkeit	-0.049
verlust	0.065
gering	-0.056
gesamt	-0.063
altersversorgung	0.076
gesundheitspolitik	-0.038
gesetz	-0.037
hartz	-0.038
perspektivlosigkeit	-0.100
abwanderung	-0.140
jugendkriminalität	-0.038
islam	0.064
west	-0.179
bundesregierung	-0.039
unterschiede	-0.075
staatsverschuldung	0.068
ständig	-0.048
preisentwicklung	-0.036
überfremdung	0.070
sozialabbau	-0.045
altersarmut	-0.041
berufsausbildung	-0.036
bundesländer	-0.144
justiz	-0.047

Results of correlational analyses of the complete sample, dependent variable: sample region (0 = East Germany, 1 = West Germany). Only words with $p < .001$ are displayed, numbers indicate semi-standardized regression coefficients

politik	-0.021
arbeitslosigkeit	-0.065
deutschland	-0.052
gesundheit	0.033
jugend	-0.050
entwicklung	-0.045
sorgen	0.030

menschen	-0.025
arbeit	-0.045
sozial	-0.035
bekommen	-0.015
hohe	-0.042
wirtschaft	-0.019
mann	0.019
sicher	-0.021
geld	-0.017
regierung	-0.029
korruption	-0.028
fehl	-0.026
staat	-0.034
reich	-0.023
enkel	-0.032
bildung	-0.020
ungerecht	-0.041
steigend	-0.038
arm	-0.025
jung	-0.025
über	-0.021
finanziell	-0.017
gesundheitsreform	-0.032
eltern	0.020
land	-0.017
bildungspolitik	-0.025
lehrstelle	-0.037
ost	-0.073
kriminalität	-0.019
mutter	0.022
usa	-0.029
lage	-0.015
gesundheitswesen	-0.041
globalisierung	-0.014
erhalt	-0.023
preise	-0.025
perspektiven	-0.037
kosten	-0.026
jugendarbeitslosigkeit	-0.023
armut	-0.015
verlust	0.020
brd	-0.017
gering	-0.025
irak	-0.014

gesamt	-0.028
bildungssystem	-0.015
altersversorgung	0.025
gesundheitspolitik	-0.018
gesetz	-0.018
hartz	-0.018
perspektivlosigkeit	-0.040
abwanderung	-0.054
jugendkriminalität	-0.018
bürokratie	-0.015
abbau	-0.015
west	-0.067
untereinander	-0.014
chancen	-0.015
bundesregierung	-0.018
unterschiede	-0.031
ständig	-0.021
betreu	-0.015
preisentwicklung	-0.017
sozialabbau	-0.020
verarmung	-0.015
altersarmut	-0.018
bezahlung	-0.015
berufsausbildung	-0.016
bildungsniveau	-0.016
bundesländer	-0.054
justiz	-0.020
kluft	-0.013

Results of correlational analyses of the subsample providing free text answers, dependent variable: educational level. Only words with $p < .001$ are displayed, numbers indicate semi-standardized regression coefficients

zukunft	-0.049
politik	0.063
arbeitslosigkeit	-0.146
nicht	-0.089
entwicklung	0.075
sorgen	-0.036
arbeit	-0.171

familie	0.056
sozial	0.082
rente	-0.100
alles	-0.079
bekommen	-0.162
gesellschaft	0.130
sohn	-0.048
finden	-0.110
arbeitsplatz	-0.076
wirtschaft	0.035
zunehmend	0.107
tochter	-0.052
mann	-0.078
ausländer	-0.082
beruflich	0.056
leben	-0.073
geld	-0.063
korruption	0.035
fehl	0.037
egoismus	0.055
enkel	-0.037
bildung	0.117
leute	-0.042
arm	0.043
euro	-0.077
krieg	-0.046
unfähigkeit	0.041
frau	-0.043
über	-0.034
bse	-0.040
eltern	0.056
werteverfall	0.101
enkelkinder	-0.049
arbeitsplätze	-0.043
moral	0.057
bildungspolitik	0.099
lehrstelle	-0.068
werte	0.073
kriminalität	-0.039
usa	0.079
umgang	0.049
lage	-0.042
bleiben	-0.063
globalisierung	0.067

verfall	0.045
preise	-0.066
perspektiven	0.033
mangelnde	0.092
angst	-0.041
medien	0.091
studium	0.090
machen	-0.053
ausbilungsplatz	-0.077
verlust	0.065
erziehung	0.062
dummheit	0.077
terrorismus	0.039
mal	-0.055
bildungssystem	0.072
haus	-0.046
demokratie	0.035
altersversorgung	0.048
weitergehen	-0.061
teurer	-0.062
arbeitslose	-0.036
ehemann	-0.043
wachsend	0.040
werteverlust	0.066
einfluss	0.068
abbau	0.036
klimawandel	0.037
schere	0.052
ehefrau	-0.041
lehre	-0.041
wohnung	-0.044
ignoranz	0.060
kultur	0.069
wohlergehen	0.034
arbeitsstelle	-0.054
intoleranz	0.040
schulpolitik	0.062
arroganz	0.034
hoffen	-0.047
bildungsniveau	0.064
studiengebühren	0.065
teuer	-0.038
steigenden	-0.032
schlimm	-0.043

straßen	-0.039
schwach	0.040
verdummung	0.053

Results of correlational analyses of the complete sample, dependent variable: educational level. Only words with $p < .001$ are displayed, numbers indicate semi-standardized regression coefficients

kinder	0.048
zukunft	0.022
politik	0.066
arbeitslosigkeit	-0.025
deutschland	0.029
gesundheit	0.019
jugend	0.034
entwicklung	0.051
menschen	0.033
arbeit	-0.043
familie	0.040
sozial	0.051
rente	-0.016
allgemein	0.022
bekommen	-0.044
gesellschaft	0.066
finden	-0.027
arbeitsplatz	-0.014
wirtschaft	0.030
ausbildung	0.023
alt	0.014
schule	0.025
zunehmend	0.056
mann	-0.014
ausländer	-0.015
situation	0.019
beruflich	0.034
regierung	0.016
korruption	0.027
fehl	0.027
reich	0.025
egoismus	0.034

bildung	0.056
arm	0.029
jung	0.013
euro	-0.017
unfähigkeit	0.028
eltern	0.031
werteverfall	0.049
welt	0.018
problem	0.016
moral	0.033
bildungspolitik	0.047
lehrstelle	-0.016
partei	0.017
werte	0.038
usa	0.040
umgang	0.028
gleichgültigkeit	0.016
gewalt	0.014
bleiben	-0.014
globalisierung	0.035
verfall	0.027
preise	-0.016
perspektiven	0.021
beruf	0.015
mangelnde	0.044
medien	0.043
bürger	0.015
bevölkerung	0.020
armut	0.015
viel	0.021
studium	0.040
ausbilungsplatz	-0.023
verlust	0.033
erziehung	0.031
dummheit	0.037
terrorismus	0.023
brd	0.020
schulbildung	0.017
mal	-0.013
versorgung	0.016
generation	0.018
verhalten	0.017
kinderfeindlichkeit	0.017
bildungssystem	0.034

stark	0.016
demokratie	0.021
altersversorgung	0.025
weitergehen	-0.016
miteinander	0.014
gesundheitspolitik	0.018
verlogenheit	0.016
manager	0.012
teurer	-0.017
privat	0.013
folgen	0.014
perspektivlosigkeit	0.017
reform	0.016
zustand	0.013
zunahme	0.016
wachsend	0.022
bush	0.016
werteverlust	0.032
einfluss	0.033
gegenüber	0.017
abbau	0.020
klimawandel	0.019
schere	0.026
beziehung	0.014
schulsystem	0.016
amerika	0.014
denken	0.013
behinderte	0.013
ignoranz	0.029
gewaltbereitschaft	0.017
kultur	0.032
wohlergehen	0.018
verrohung	0.017
staatsverschuldung	0.017
arbeitsstelle	-0.015
intoleranz	0.021
schulpolitik	0.028
politikverdrossenheit	0.018
überfremdung	0.016
kinderbetreuung	0.016
mitmenschen	0.015
missbrauch	0.016
verarmung	0.013
europa	0.017

kalt	0.015
bezug	0.014
ehrlich	0.013
arroganz	0.018
bildungsniveau	0.029
studiengebühren	0.028
justiz	0.014
kluft	0.018
nah	0.016
sitten	0.012
schwach	0.020
verdummung	0.024

Results of correlational analyses of the subsample providing free text answers, dependent variable: life satisfaction. Only words with $p < .001$ are displayed, numbers indicate standardized regression coefficients

kinder	0.030
zukunft	-0.018
arbeitslosigkeit	-0.042
nicht	-0.031
jugend	0.033
sorgen	0.021
arbeit	-0.062
rente	-0.026
alles	-0.034
bekommen	-0.036
gesellschaft	0.025
sohn	-0.020
finden	-0.040
arbeitsplatz	-0.028
ausbildung	0.019
alt	-0.019
schule	0.034
mann	-0.037
situation	-0.023
leben	-0.044
geld	-0.040
staat	-0.023
egoismus	0.024

bildung	0.029
ungerecht	-0.026
steigend	-0.018
euro	-0.020
frau	-0.025
finanziell	-0.031
gesundheitsreform	-0.018
bse	0.018
eltern	0.020
werteverfall	0.033
moral	0.026
bildungspolitik	0.020
krankheit	-0.031
werte	0.024
gesundheitswesen	-0.038
verfall	0.020
mangelnde	0.021
kosten	-0.021
medien	0.019
erziehung	0.018
schulbildung	0.018
gering	-0.018
person	-0.018
generation	0.020
krank	-0.026
haus	-0.018
einkommen	-0.021
weitergehen	-0.029
hartz	-0.022
werteverlust	0.023
einfluss	0.025
rentner	-0.023
schulsystem	0.017
wohnung	-0.029
wohlergehen	0.019
schwer	-0.023
arbeitsstelle	-0.018
schulpolitik	0.021
politikverdrossenheit	0.019
missbrauch	0.020
altersarmut	-0.020
bezahlung	-0.023
bildungsniveau	0.021
religion	0.020

partnerschaft	-0.022

Results of correlational analyses of the complete sample, dependent variable: life satisfaction.

Only words with $p < .001$ are displayed, numbers indicate standardized regression coefficients

kinder	-0.007
zukunft	-0.022
politik	-0.008
arbeitslosigkeit	-0.026
deutschland	-0.014
nicht	-0.020
arbeit	-0.033
sozial	-0.012
rente	-0.016
alles	-0.020
bekommen	-0.022
sohn	-0.013
hohe	-0.007
finden	-0.023
arbeitsplatz	-0.018
alt	-0.012
schule	0.007
tochter	-0.007
mann	-0.019
ausländer	-0.007
schlecht	-0.007
situation	-0.015
leben	-0.022
geld	-0.020
regierung	-0.010
korruption	-0.008
staat	-0.014
reich	-0.008
bildung	0.007
ungerecht	-0.014
steigend	-0.011
arm	-0.007
euro	-0.012
frau	-0.013
finanziell	-0.016
gesundheitsreform	-0.010

werteverfall	0.009
moral	0.007
krankheit	-0.016
ost	-0.007
lage	-0.007
gesundheitswesen	-0.018
preise	-0.008
angst	-0.007
kosten	-0.011
mal	-0.007
gering	-0.010
versorgung	-0.008
person	-0.010
krank	-0.012
haus	-0.009
arbeitsmarkt	-0.007
gesundheitszustand	-0.008
einkommen	-0.011
weitergehen	-0.014
teurer	-0.009
privat	-0.008
hartz	-0.011
gerechtigkeit	-0.007
werteverlust	0.007
einfluss	0.008
rentner	-0.010
west	-0.007
drogen	-0.007
wohnung	-0.014
menschlich	-0.007
job	-0.007
schwer	-0.011
arbeitsstelle	-0.010
ständig	-0.008
preisentwicklung	-0.007
suchen	-0.008
verarmung	-0.008
altersarmut	-0.010
bezahlung	-0.011
bildungsniveau	0.007
teuer	-0.008
trotz	-0.006
pflege	-0.007
partnerschaft	-0.011

weiß	-0.007

Results of correlational analyses of the subsample providing free text answers, dependent variable: extraversion. Only words with $p < .001$ are displayed, numbers indicate standardized regression coefficients

arbeitslosigkeit	-0.020
gesundheit	-0.022
arbeit	-0.019
ausländer	0.029
angst	-0.019
wachsend	-0.020
staatsverschuldung	-0.022
überfremdung	0.017
religion	0.024

Results of correlational analyses of the complete sample, dependent variable: extraversion. Only words with $p < .001$ are displayed, numbers indicate standardized regression coefficients

kinder	0.009
zukunft	0.007
politik	0.016
deutschland	0.011
jugend	0.011
entwicklung	0.008
menschen	0.011
allgemein	0.009
ausländer	0.014
deutsch	0.009
regierung	0.009
über	0.009
gesundheitsreform	0.007
moral	0.008
lehrstelle	0.007
partei	0.009
kriminalität	0.008
uns	0.008

miteinander	0.007
manager	0.008
etc	0.007
staatsverschuldung	-0.007
überfremdung	0.008
religion	0.010

Results of correlational analyses of the subsample providing free text answers, dependent variable: emotional stability. Only words with $p < .001$ are displayed, numbers indicate standardized regression coefficients

kinder	-0.048
zukunft	-0.052
politik	0.027
gesundheit	-0.027
arbeit	-0.032
familie	-0.026
sohn	-0.026
alt	-0.018
tochter	-0.027
egoismus	0.018
krankheit	-0.019
mangelnde	0.020
versorgung	-0.022
werteverlust	0.022
west	-0.020
politikverdrossenheit	0.021
bildungsniveau	0.017
religion	0.022

Results of correlational analyses of the complete sample, dependent variable: emotional stability. Only words with $p < .001$ are displayed, numbers indicate standardized regression coefficients

kinder	-0.030
zukunft	-0.030

arbeitslosigkeit	-0.012
gesundheit	-0.017
nicht	-0.008
entwicklung	-0.010
arbeit	-0.018
familie	-0.016
sozial	-0.008
rente	-0.011
bekommen	-0.009
sohn	-0.014
ausbildung	-0.009
alt	-0.011
tochter	-0.014
mann	-0.010
situation	-0.007
leben	-0.009
enkel	-0.006
ungerecht	-0.007
gesundheitsreform	-0.006
enkelkinder	-0.009
krankheit	-0.010
gesundheitswesen	-0.007
beruf	-0.008
angst	-0.009
studium	-0.008
versorgung	-0.010
werteverlust	0.007
west	-0.009
wohnung	-0.007
menschlich	-0.008
preisentwicklung	-0.007
religion	0.007
weiß	-0.007

Results of correlational analyses of the subsample providing free text answers, dependent variable: agreeableness. Only words with $p < .001$ are displayed, numbers indicate standardized regression coefficients

politik	-0.028
arbeitslosigkeit	0.026

bekommen	0.026
finden	0.019
arbeitsplatz	0.037
deutsch	-0.019
regierung	-0.020
frau	0.022
gesundheitsreform	0.024
dummheit	-0.030
tierschutz	-0.028
überfremdung	-0.018

Results of correlational analyses of the complete sample, dependent variable: agreeableness. Only words with $p < .001$ are displayed, numbers indicate standardized regression coefficients

politik	-0.011
arbeitslosigkeit	0.008
bekommen	0.008
arbeitsplatz	0.012
deutsch	-0.008
regierung	-0.008
frau	0.008
gesundheitsreform	0.008
dummheit	-0.011
gesamt	-0.007
hartz	-0.007
tierschutz	-0.011
überfremdung	-0.007

Results of correlational analyses of the subsample providing free text answers, dependent variable: conscientiousness. Only words with $p < .001$ are displayed, numbers indicate standardized regression coefficients

deutschland	0.026
nicht	0.024
bekommen	0.021
sohn	0.021
arbeitsplatz	0.024

ausländer	0.027
staat	0.022
bildung	-0.023
land	0.027
kosten	0.018
medien	-0.023
studium	-0.019
besser	0.018

Results of correlational analyses of the complete sample, dependent variable: conscientiousness. Only words with $p < .001$ are displayed, numbers indicate standardized regression coefficients

arbeitslosigkeit	0.008
deutschland	0.010
nicht	0.010
bekommen	0.008
sohn	0.009
arbeitsplatz	0.009
ausländer	0.010
staat	0.008
bildung	-0.008
land	0.010
kosten	0.007
medien	-0.008
studium	-0.007
besser	0.007

Results of correlational analyses of the subsample providing free text answers, dependent variable: openness to experience. Only words with $p < .001$ are displayed, numbers indicate standardized regression coefficients

kinder	-0.022
politik	0.035
arbeitslosigkeit	-0.049
gesundheit	-0.041
nicht	-0.025
jugend	0.019

entwicklung	0.020
menschen	0.038
arbeit	-0.053
sozial	0.019
rente	-0.031
allgemein	0.019
gesellschaft	0.041
hohe	-0.022
finden	-0.033
zunehmend	0.023
mann	-0.034
egoismus	0.023
bildung	0.032
frau	-0.032
werteverfall	0.029
arbeitsplätze	-0.019
moral	0.027
bildungspolitik	0.034
werte	0.026
bleiben	-0.024
verfall	0.022
preise	-0.027
mangelnde	0.026
angst	-0.023
medien	0.030
verlust	0.022
dummheit	0.020
mal	-0.020
haus	-0.022
ehemann	-0.024
werteverlust	0.031
schere	0.019
wohnung	-0.022
kultur	0.029
intoleranz	0.022
tiere	0.021
ehrlich	0.021
arroganz	0.017
schwach	0.019

Results of correlational analyses of the complete sample, dependent variable: openness to experience. Only words with $p < .001$ are displayed, numbers indicate standardized regression coefficients

kinder	0.022
zukunft	0.020
politik	0.039
deutschland	0.024
nicht	0.007
jugend	0.022
entwicklung	0.022
sorgen	0.011
menschen	0.028
familie	0.008
sozial	0.020
alles	0.007
allgemein	0.019
gesellschaft	0.026
sohn	0.008
wirtschaft	0.012
ausbildung	0.008
alt	0.008
schule	0.014
zunehmend	0.018
ausländer	0.007
situation	0.009
beruflich	0.012
deutsch	0.014
leben	0.007
regierung	0.008
korruption	0.012
fehl	0.010
staat	0.009
reich	0.011
egoismus	0.017
bildung	0.020
arm	0.010
unfähigkeit	0.010
über	0.009
eltern	0.008
werteverfall	0.018
enkelkinder	0.008

welt	0.012
land	0.010
moral	0.017
bildungspolitik	0.019
partei	0.010
ost	0.008
werte	0.016
kriminalität	0.010
usa	0.012
umgang	0.012
gleichgültigkeit	0.012
uns	0.010
gewalt	0.007
globalisierung	0.013
verfall	0.014
mangelnde	0.015
jugendarbeitslosigkeit	0.007
medien	0.017
bürger	0.007
bevölkerung	0.010
viel	0.008
studium	0.008
verlust	0.013
erziehung	0.011
dummheit	0.013
terrorismus	0.010
brd	0.010
schulbildung	0.009
generation	0.009
volk	0.009
kinderfeindlichkeit	0.010
bildungssystem	0.009
demokratie	0.010
altersversorgung	0.009
miteinander	0.011
gesundheitspolitik	0.007
verlogenheit	0.009
manager	0.007
frieden	0.007
folgen	0.008
perspektivlosigkeit	0.007
gerechtigkeit	0.008
wachsend	0.008
bush	0.009

werteverlust	0.016
jugendkriminalität	0.008
einfluss	0.009
bürokratie	0.007
gegenüber	0.008
tierschutz	0.008
klimawandel	0.007
schere	0.011
rentner	0.007
etc	0.008
schulsystem	0.007
umwelt	0.011
denken	0.008
drogen	0.008
ignoranz	0.009
menschlich	0.009
gewaltbereitschaft	0.008
kultur	0.015
wohlergehen	0.007
untereinander	0.009
verrohung	0.009
intoleranz	0.012
schulpolitik	0.007
politikverdrossenheit	0.009
überfremdung	0.008
europa	0.008
tiere	0.011
kalt	0.010
zuwanderung	0.007
ehrlich	0.011
arroganz	0.010
studiengebühren	0.007
negativ	0.008
studienplatz	0.007
kluft	0.008
brutalität	0.008
kindererziehung	0.008
schwach	0.010
verdummung	0.009
religion	0.008

Results of correlational analyses of the subsample providing free text answers, dependent variable: openness to experience, controlled for education. Only words with $p < .001$ are displayed, numbers indicate standardized regression coefficients

kinder	-0.020
politik	0.024
arbeitslosigkeit	-0.031
gesundheit	-0.035
menschen	0.033
arbeit	-0.028
rente	-0.019
gesellschaft	0.024
hohe	-0.019
mann	-0.023
frau	-0.025
moral	0.020
bildungspolitik	0.024
angst	-0.019
werteverlust	0.023
kultur	0.026
staatsverschuldung	-0.022
tiere	0.020
ehrlich	0.018

Results of correlational analyses of the complete sample, dependent variable: openness to experience, controlled for education. Only words with $p < .001$ are displayed, numbers indicate standardized regression coefficients

kinder	0.018
zukunft	0.018
politik	0.029
deutschland	0.020
nicht	0.008
jugend	0.018
entwicklung	0.016
sorgen	0.013
menschen	0.023
sozial	0.014

alles	0.007
allgemein	0.016
bekommen	0.010
gesellschaft	0.019
sohn	0.008
wirtschaft	0.010
schule	0.011
zunehmend	0.012
ausländer	0.008
situation	0.008
beruflich	0.008
deutsch	0.012
regierung	0.008
korruption	0.009
fehl	0.009
staat	0.008
reich	0.008
egoismus	0.013
bildung	0.013
über	0.008
werteverfall	0.012
enkelkinder	0.009
welt	0.009
land	0.008
moral	0.013
bildungspolitik	0.015
lehrstelle	0.008
partei	0.009
werte	0.012
kriminalität	0.009
usa	0.009
umgang	0.008
gleichgültigkeit	0.010
uns	0.009
globalisierung	0.009
verfall	0.011
mangelnde	0.011
jugendarbeitslosigkeit	0.007
medien	0.011
bevölkerung	0.008
verlust	0.009
erziehung	0.009
dummheit	0.009
terrorismus	0.007

brd	0.007
schulbildung	0.007
generation	0.007
volk	0.010
kinderfeindlichkeit	0.008
demokratie	0.007
miteinander	0.009
verlogenheit	0.007
werteverlust	0.012
jugendkriminalität	0.007
tierschutz	0.007
schere	0.008
umwelt	0.009
drogen	0.009
menschlich	0.008
kultur	0.013
untereinander	0.007
verrohung	0.007
intoleranz	0.009
überfremdung	0.006
tiere	0.010
kalt	0.010
ehrlich	0.010
arroganz	0.008
negativ	0.007
brutalität	0.007
schwach	0.008
